# Self-assembled polyoxometalate enables efficient and stable proton conduction in high-temperature polymer electrolyte membranes

**DOI:** 10.1039/d6sc05807h

**Published:** 2026-08-26

**Authors:** Tingting Li, Yi Zhang, Peng Zuo, Shihao Song, Rong-Lin Zhong, Haolong Li

**Affiliations:** a State Key Laboratory of Supramolecular Structure and Materials, College of Chemistry, Jilin University Changchun 130012 China hl_li@jlu.edu.cn

## Abstract

High-temperature proton exchange membrane fuel cells (HT-PEMFCs) are attractive for their high fuel-impurity tolerance and simplified water/thermal management, but their performance is often limited by phosphoric acid (PA) loss from the membrane, especially below 100 °C, where condensed water accelerates PA leaching. Here we report a self-assembled polyoxometalate (POM) strategy to immobilize PA and promote stable PA-mediated proton conduction. A polyethylene glycol-grafted POM (GSiW_11_) was designed and co-incorporated with PA into a poly(terphenyl piperidinium) (PTP) matrix to prepare hybrid high-temperature proton exchange membranes (HT-PEMs). GSiW_11_ plays two important roles. At the molecular level, it forms supramolecular complexes with PA, lowering the proton dissociation energy of PA and facilitating proton transport. At the nanoscale, it induces microphase separation of PTP to generate continuous ionic domains that confine PA and construct stable proton-conducting networks. The resulting hybrid membrane exhibits a high proton conductivity of 91 mS cm^−1^ at 180 °C with a relatively low PA uptake of 125%. The corresponding H_2_/O_2_ fuel cell delivers a peak power density of 1130 mW cm^−2^ at 160 °C and operates stably over a broad temperature range of 80–160 °C. This work demonstrates the potential of self-assembled POMs as functional additives for designing HT-PEMs with efficient and stable proton-conduction performance.

## Abstract 2

Self-assembled polyoxometalates induce phosphoric-acid-confined ionic domains in a poly(terphenyl piperidinium) matrix and form synergistic proton-conducting complexes, enabling efficient proton transport and stable phosphoric acid immobilization.

## Graphical abstract

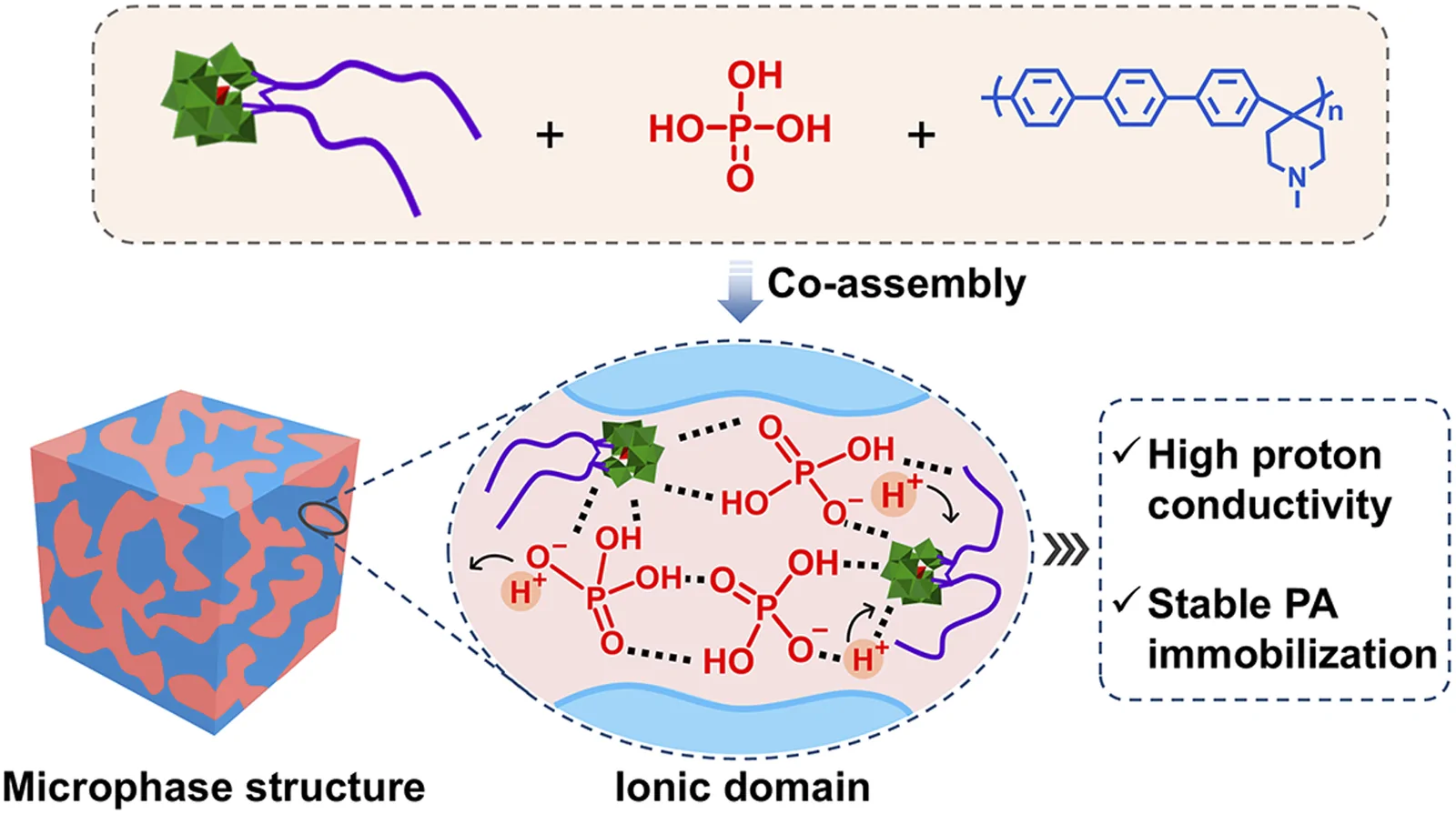

## Introduction

Fuel cells are clean and efficient energy conversion devices and represent an important solution for building sustainable energy systems.^1–3^ Among various fuel cells, HT-PEMFCs have attracted broad attention owing to their fast electrode reaction kinetics, high carbon monoxide tolerance, and simplified water and thermal management.^4,5^ The core component of HT-PEMFCs is the high-temperature proton exchange membrane (HT-PEM), which is typically based on phosphoric acid (PA)-doped polymer electrolytes.^6,7^ In these membranes, anhydrous proton conduction at elevated temperatures relies on hydrogen-bonding networks formed by PA molecules.^8–10^ However, because PA has high water solubility and mobility, it is prone to leaching during fuel cell operation.^11,12^ This problem becomes especially severe when the cell operates across temperatures below 100 °C, where condensed water accelerates PA dissolution and leaching, leading to decreased proton conductivity and rapid performance decay.^13,14^ Therefore, improving PA retention has become a key challenge for advancing the practical application of HT-PEMFCs.

Several strategies have been developed to enhance PA retention in HT-PEMs. Among them, introducing additives capable of complexing with PA to construct synergistic proton-conducting systems has proven effective.^15–18^ These additives usually contain abundant basic sites that bind PA through supramolecular interactions such as hydrogen bonding or electrostatic interactions, while also promoting PA proton dissociation and transport. For example, Lu and co-workers introduced ethylenediamine tetramethylene phosphonic acid into a membrane matrix to construct a “dual proton conductor” system with PA, achieving efficient and stable proton conduction.^12^ Hu and co-workers incorporated a quaternized porous aromatic framework into sulfonated polybenzimidazole, where quaternary ammonium groups served as basic sites to anchor PA and formed ionic cross-linking networks with sulfonic acid groups, thereby significantly enhancing PA retention and proton conductivity.^16^ In addition, constructing microphase-separated structures in membranes can confine PA within hydrophilic domains and generate continuous nanoscale proton-transport channels, which effectively improves PA retention and lowers the proton-transport energy barrier.^19–22^ Notably, strategies that integrate molecular complexation with microphase confinement to synergistically improve PA retention remain limited. This requires multifunctional additives that can both form stable complexes with PA and induce microphase separation within the membrane.

Polyoxometalates (POMs) are molecularly defined metal–oxide nanoclusters with typical sizes of approximately 1 nm.^23,24^ They possess tunable structures and diverse functionalities and have been widely used as functional building blocks for hybrid materials.^25–32^ POMs exhibit excellent proton-conducting ability because their oxygen-rich surfaces can form hydrogen-bonding networks with surrounding polar molecules containing nitrogen or oxygen atoms, including PA.^22,33^ These networks provide continuous proton-hopping pathways, enabling high proton conductivity under low-humidity or even anhydrous conditions. These features make POMs promising candidates for the fabrication of proton exchange membranes. Moreover, POMs can co-assemble with polymers to form POM-enriched nanoscale domains.^26,34^ In our previous work, we constructed three-dimensionally continuous POM-based proton-transport channels in polymer matrices by co-assembling POMs with polymers of different topologies, including block copolymers and comb-like polymers.^35–38^ In addition, rational surface modification of POMs, such as grafting organic groups onto their surfaces, can impart amphiphilic features and further promote their self-assembly.^39–42^ Based on these characteristics, POMs are well suited as self-assembling molecular additives for developing highly conductive HT-PEMs in combination with PA.

In this work, we designed and synthesized a polyethylene glycol (PEG)-grafted POM, GSiW_11_, and co-incorporated it with PA into a poly(terphenyl piperidinium) (PTP) matrix to prepare hybrid HT-PEMs. GSiW_11_ forms supramolecular complexes with PA as synergistic proton conductors, thereby lowering the proton dissociation energy of PA. Meanwhile, GSiW_11_ self-assembles into nanoscale ionic domains within the PTP matrix, confining PA in these domains to construct stable proton-conducting networks, as illustrated in Fig. 1. Benefiting from these effects, the resulting hybrid membranes exhibit high proton conductivity, improved PA retention, and enhanced mechanical strength at relatively low PA doping levels, enabling their fuel cells to operate stably over a broad temperature range of 80–160 °C. We systematically investigate the supramolecular complexation between GSiW_11_ and PA, elucidate the synergistic proton-conduction mechanism, and clarify how membrane microstructure correlates with macroscopic proton-conducting and mechanical properties. This work presents a strategy for using self-assembled POMs to immobilize PA and facilitate PA-mediated proton conduction, providing a new solution for developing HT-PEMs with stable proton-conduction performance.

**Fig. 1:**
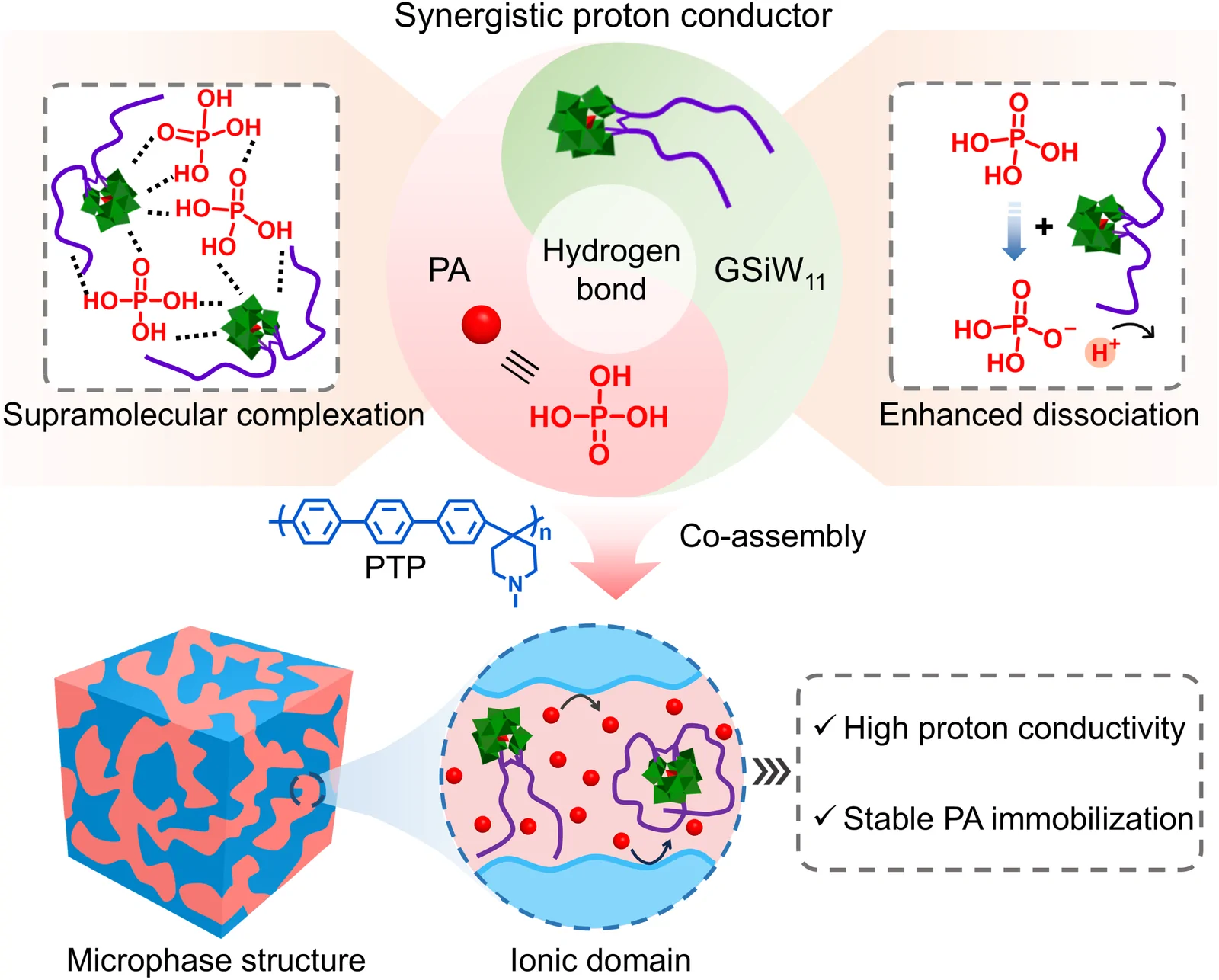
Schematic illustration of the synergistic effects of GSiW_11_, which (i) forms synergistic proton conductors with PA at the molecular level and (ii) induces PTP to form microphase-separated structures at the nanoscale.

## Results and discussions

### Design and synthesis of GSiW_11_ and PTP

Recent studies have shown that PEG-grafted POMs exhibit rich self-assembly behavior.^39^ Meanwhile, ether oxygen groups on PEG chains can cooperate with PA through hydrogen-bonding interactions to facilitate proton conduction.^22,33^ Moreover, we recently found that grafting PEG chains with appropriate lengths onto POMs can generate a superchaotropic effect, which substantially decreases the water solubility of POMs and enables their stable immobilization in Nafion membranes without dissolution or leaching.^38,43^ Based on these considerations, PEG was covalently grafted onto Keggin-type K_8_[SiW_11_O_39_] (SiW_11_) through stable Si–O–Si bonds to afford PEG-grafted POMs (GSiW_11_) as the designed additive, as shown in Scheme S1. The FTIR spectrum of GSiW_11_ displays a characteristic absorption peak at 1043 cm^−1^ corresponding to Si–O–Si bonds, confirming the successful grafting reaction (Fig. S1).^43^ Meanwhile, the characteristic W–O vibration peaks of the SiW_11_ cluster are retained, indicating that the cluster framework remains intact.^32^^1^H NMR analysis shows that the average degree of polymerization of the PEG chains in GSiW_11_ is 10 (Fig. S2). Combined with elemental analysis, the chemical formula of GSiW_11_ is determined to be K_4_[SiW_11_O_39_(SiC_2*n*+4_H_4*n*+9_O_*n*+1_)_2_O]. Inductively coupled plasma spectroscopy (ICP) confirms that the K^+^ counterions of GSiW_11_ are completely replaced by H^+^ counterions (see the SI). In addition, thermogravimetric analysis (TGA) shows no obvious weight loss of GSiW_11_ below 200 °C (Fig. S3), indicating excellent thermal stability suitable for the high-temperature operating environment of HT-PEMFCs.

Poly(terphenyl piperidinium) (PTP) is one of the most synthetically mature and low-cost poly(arylene alkane)s.^44,45^ Its all-carbon aromatic backbone provides excellent mechanical properties, chemical stability, and thermal stability, making it widely used in polymer electrolyte membranes, particularly for HT-PEMs.^46^ The piperidine groups in PTP can serve as PA adsorption sites and possess good chemical affinity with POMs. Therefore, PTP was selected as the matrix material for HT-PEMs in this work. PTP was synthesized through superacid-catalyzed polymerization (Scheme S2 and Fig. S4). Because PTP adsorbs onto the chromatographic column, no elution peak was observed in gel permeation chromatography (GPC). Therefore, intrinsic viscosity was used to estimate its molecular weight. The intrinsic viscosity of PTP was 3.85 dL g^−1^, indicating a high molecular weight. TGA results show no obvious weight loss below 200 °C (Fig. S3), confirming the excellent thermal stability of PTP and its suitability for typical HT-PEMFC operating temperatures.

### Interactions between GSiW_11_ and PA

The surface of GSiW_11_ contains highly dissociable protons and densely packed oxygen atoms, which can act as hydrogen-bond donors and acceptors, respectively, to form hydrogen-bonding networks with PA. This not only strengthens PA complexation but also provides abundant proton-hopping pathways. Pure PA is crystalline at room temperature because of intermolecular hydrogen bonding. After mixing 2 wt% GSiW_11_ with PA, the crystallization of PA is suppressed and the system becomes liquid, indicating that GSiW_11_ participates in and reconstructs the hydrogen-bonding network of PA (Fig. 2a).

**Fig. 2:**
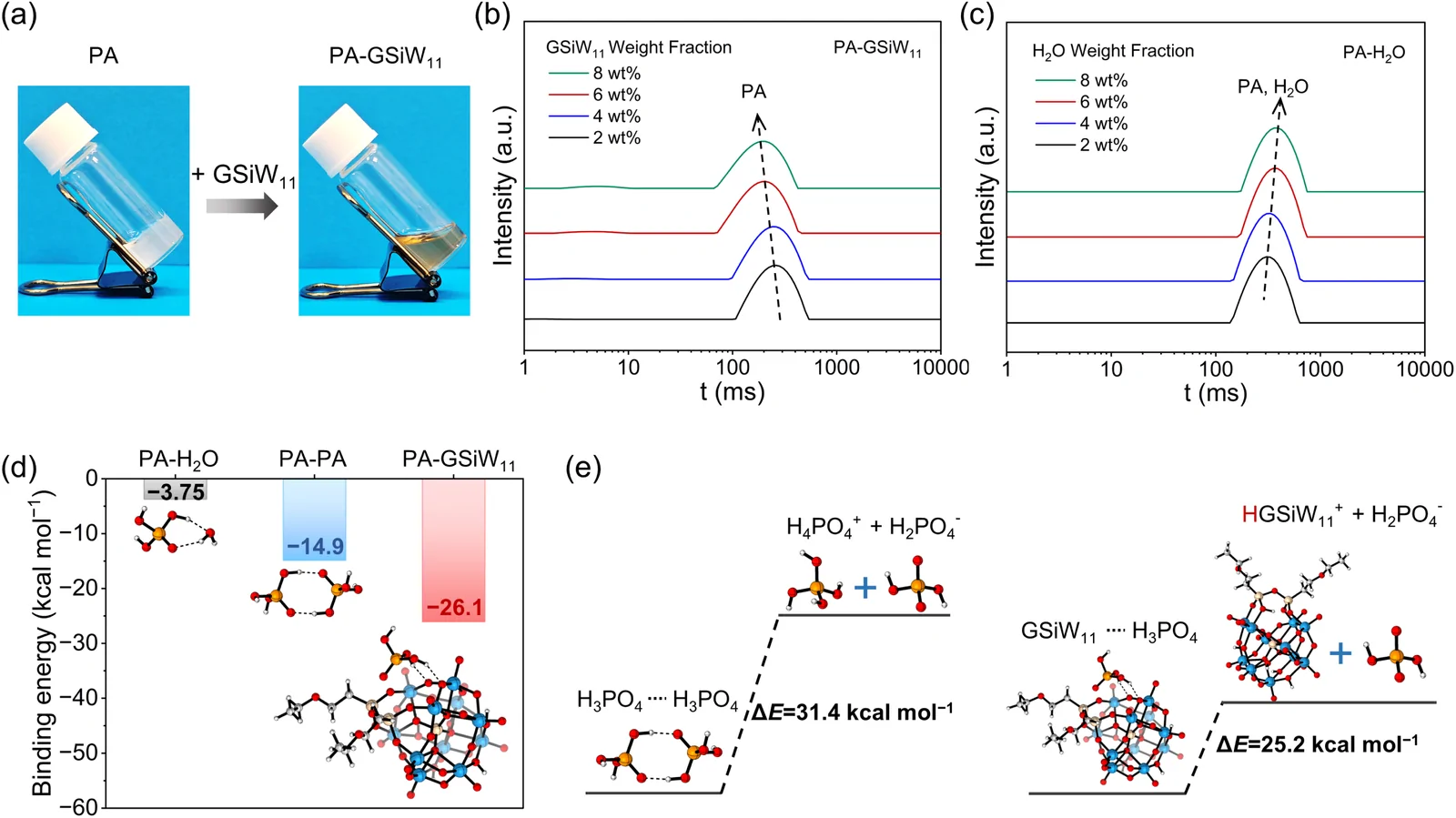
(a) Photographs of pure PA and PA–GSiW_11_ complexes containing 2 wt% GSiW_11_. *T*_2_ relaxation time distributions of pure PA mixed with (b) GSiW_11_ and (c) H_2_O. (d) Binding energies of PA–H_2_O, PA–PA, and PA–GSiW_11_ complexes. (e) Proton dissociation energies of PA and PA complexed with GSiW_11_.

Low-field nuclear magnetic resonance (LF-NMR) was used to investigate the complexation between GSiW_11_ and PA. A longer *T*_2_ relaxation time generally indicates higher molecular mobility.^47,48^ In the PA–GSiW_11_ mixed system, the *T*_2_ peak of PA gradually shifts toward shorter relaxation times as the GSiW_11_ content increases. In contrast, in the PA–H_2_O mixed system, the *T*_2_ peak shifts toward longer relaxation times with increasing H_2_O content (Fig. 2b and c). These results indicate that GSiW_11_ effectively complexes with PA and reduces its mobility, whereas H_2_O increases PA mobility.

To further understand the complexation mechanism between GSiW_11_ and PA, a GSiW_11_ model containing one PEG repeat unit was used for density functional theory (DFT) calculations. The binding energy between GSiW_11_ and PA is calculated to be −26.1 kcal mol^−1^, much stronger than that of PA–PA (−14.9 kcal mol^−1^) and PA–H_2_O (−3.75 kcal mol^−1^) interactions (Fig. 2d). This confirms the formation of stronger hydrogen-bonding interactions between GSiW_11_ and PA. Such interactions can suppress PA loss and facilitate proton transport.

The proton dissociation ability of PA is one of the key factors governing the proton conductivity of HT-PEMs. To investigate the effect of GSiW_11_ on PA proton dissociation, the dissociation energy of PA was calculated by DFT (Fig. 2e). In the pure PA system, the energy required for PA to dissociate a proton (H^+^) and form an ion pair (H_2_PO_4_^−^/H_4_PO_4_^+^) is 31.4 kcal mol^−1^. This process can induce continuous proton transfer and lead to the formation of Grotthuss chains.^8^ In the GSiW_11_–PA complex system, the dissociation energy of PA decreases to 25.2 kcal mol^−1^, indicating that GSiW_11_ effectively promotes PA proton dissociation. We propose that GSiW_11_ facilitates PA proton dissociation through two effects. First, the formation of P–O–H⋯O hydrogen bonds polarizes the O–H bond in PA, making it easier to cleave. Second, the highly delocalized negative charge of GSiW_11_ results in a low proton affinity of H_3_[SiW_11_O_39_(SiC_2*n*+4_H_4*n*+9_O_*n*+1_)_2_O]^−^, lower than those of H_2_PO_4_^−^, HSO_4_^−^, and CF_3_SO_3_^−^ (Fig. S8 and Table S1), reflecting relatively weak binding of the surface protons of GSiW_11_ and enabling these active protons to interact with H_2_PO_4_^−^ and thereby stabilize the H_2_PO_4_^−^ species through proton sharing.^49,50^

### Microphase structure of hybrid membranes

Both PTP and GSiW_11_ are readily soluble in polar solvents such as *N*-methyl-2-pyrrolidone, allowing the facile preparation of their hybrid membranes through solution blending and casting. By adjusting the mass ratio of PTP to GSiW_11_, a series of hybrid membranes were obtained and denoted as P–GSiW_11_–*x*, where *x* = 4, 8, and 12 represents the mass fraction of GSiW_11_. After introducing GSiW_11_, the resulting hybrid membranes remain homogeneous and transparent without macroscopic phase separation (Fig. 3a and S9).

**Fig. 3:**
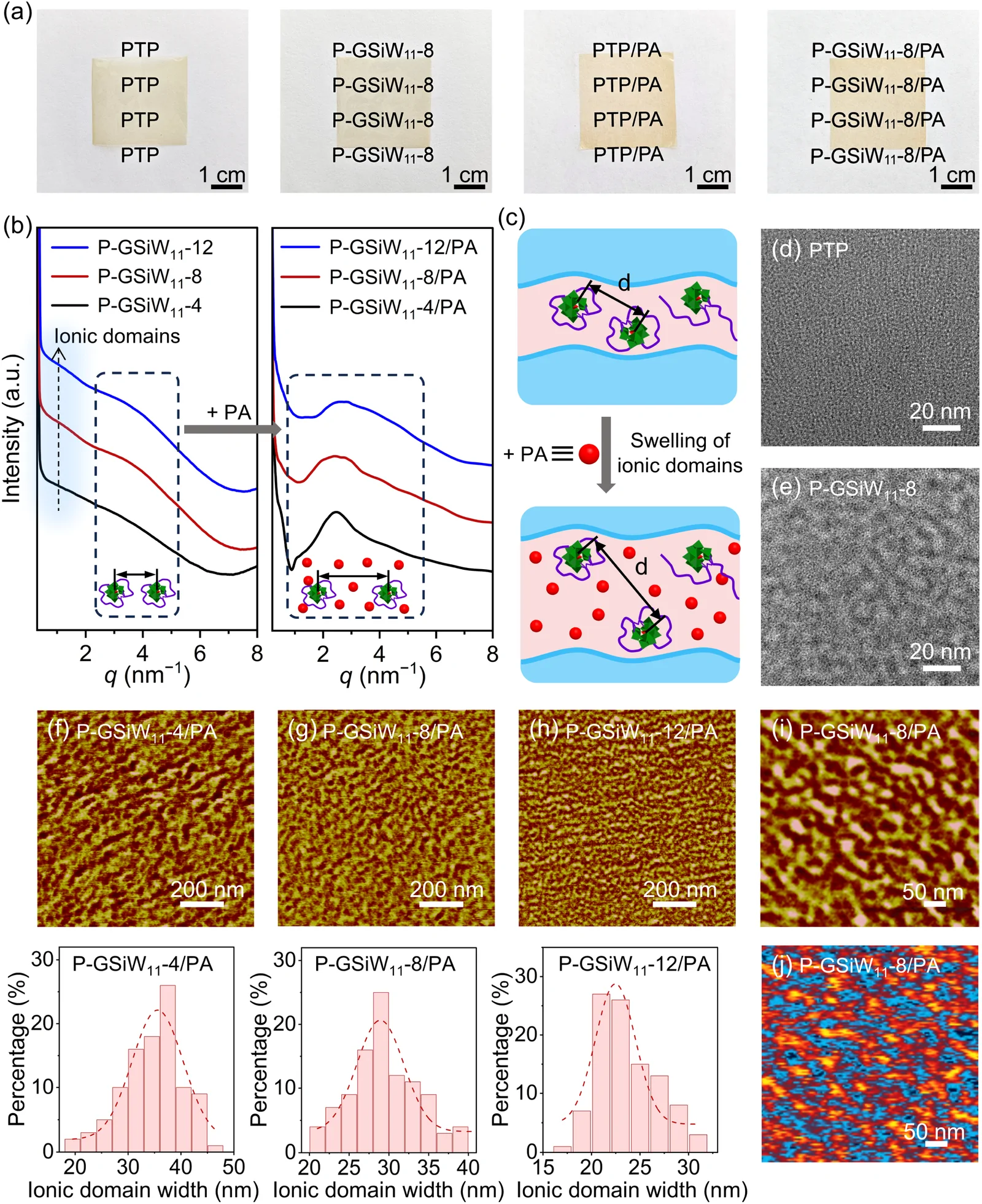
(a) Photographs of PTP, P–GSiW_11_–8, PTP/PA, and P–GSiW_11_–8/PA membranes. (b) SAXS profiles of hybrid membranes before (left) and after (right) PA doping. (c) Schematic illustration of ionic domains before and after PA doping. (d and e) TEM images of ultrathin sections of PTP and P–GSiW_11_–8 membranes. (f–h) AFM phase images of thin-sectioned P–GSiW_11_–*x*/PA membranes (*x* = 4, 8, and 12) and the corresponding ionic-domain size distributions. AFM-IR compositional mapping of the P–GSiW_11_–8/PA membrane: (i) AFM topographic image and (j) AFM-IR absorption at 950 cm^−1^.

Differential scanning calorimetry (DSC) and X-ray diffraction (XRD) were used to investigate the dispersion state of GSiW_11_ in the PTP matrix. PTP shows no glass transition before thermal decomposition, whereas GSiW_11_ displays a glass transition temperature (*T*_g_) of approximately 87 °C (Fig. S10). Notably, the DSC curves of the hybrid membranes show no characteristic *T*_g_ peak of GSiW_11_, indicating that GSiW_11_ is uniformly dispersed in the hybrid system and does not decrease the thermal stability of the membranes.^32,51^ XRD results further confirm the dispersion state of GSiW_11_ in the membrane matrix (Fig. S11). The hybrid membranes do not show diffraction peaks characteristic of aggregated GSiW_11_, indicating molecular-level dispersion of GSiW_11_ within the membranes.^32,43^ Only a broad diffraction peak at 2*θ* = 11–28° is observed, consistent with the PTP matrix and corresponding to interchain packing of PTP. Attenuated total reflection-Fourier transform infrared (ATR-FTIR) was used to further investigate the noncovalent interactions between the two components in the hybrid membranes. Compared with pristine PTP, the C–N stretching vibration peak in the hybrid membrane shifts from 1119 to 1114 cm^−1^ (Fig. S12), indicating the formation of electrostatic and hydrogen-bonding interactions between GSiW_11_ and PTP.^52^ These interactions improve the compatibility between GSiW_11_ and PTP.

Small-angle X-ray scattering (SAXS), transmission electron microscopy (TEM), and atomic force microscopy (AFM) were used to investigate the microphase-separated structures of hybrid membranes. The pristine PTP membrane shows no scattering peak in the SAXS profile, indicating the absence of nanoscale phase separation (Fig. S13).^22^ After introducing GSiW_11_, the hybrid membranes display distinct scattering peaks at 0.9 and 3.6 nm^−1^, corresponding to structural sizes of approximately 7.0 and 1.7 nm, respectively. The intensities of these peaks increase with increasing GSiW_11_ content, indicating that GSiW_11_ induces microphase separation in the hybrid membranes. The peak at 0.9 nm^−1^ reflects the ionic domains formed by GSiW_11_ and the piperidine groups of PTP, whereas the peak at 3.6 nm^−1^ corresponds to inter-cluster scattering between adjacent GSiW_11_ clusters within the domains (Fig. 3b).^22,53^ To clarify the role of GSiW_11_ in inducing PTP microphase separation, a hybrid membrane containing 8 wt% H_4_SiW_12_O_40_ (P–SiW_12_–8) was prepared as a control. However, this membrane shows no scattering signal corresponding to ionic domains, strongly demonstrating that PEG grafting endows GSiW_11_ with the ability to induce PTP self-assembly into microphase-separated structures, whereas unmodified bare H_4_SiW_12_O_40_ clusters do not have this ability (Fig. S14). This is likely because PEG grafting imparts amphiphilicity to GSiW_11_ and enhances its self-assembly capability.^39^

After further doping the P–GSiW_11_–*x* membranes with PA, the resulting P–GSiW_11_–*x*/PA hybrid membranes remain homogeneous and transparent but show a certain degree of swelling. SAXS results show that all scattering peaks shift toward lower *q* values after PA doping (Fig. 3b). As a result, the scattering peak corresponding to the ionic domains shifts to *q* values below 0.3 nm^−1^, beyond the detection range.^22^ Meanwhile, the scattering peak corresponding to the inter-cluster distance of GSiW_11_ within the ionic domains shifts to approximately 2.5 nm^−1^, indicating that the inter-cluster distance increases to approximately 2.5 nm. These results suggest that PA enters the ionic domains of the membranes and swells these domains.

TEM results further confirm the microphase-separated structures of these membranes (Fig. 3d and e). The pristine PTP membrane shows a homogeneous morphology, whereas the P–GSiW_11_–8 hybrid membrane exhibits a typical microphase-separated structure. The dark regions correspond to ionic domains formed by GSiW_11_ and piperidine groups with a size of approximately 7 nm, whereas the bright regions correspond to the hydrophobic phase formed by the PTP backbone, which provides mechanical support.^31^ AFM was used to investigate the microphase-separated structures of the PA-doped membranes (Fig. 3f–h). After PA doping, the pristine PTP/PA membrane retains a homogeneous structure (Fig. S15), whereas the ionic domains in the hybrid membranes are significantly enlarged. The dark regions correspond to ionic domains formed by PA, GSiW_11_, and piperidine groups. From P–GSiW_11_–4/PA and P–GSiW_11_–8/PA to P–GSiW_11_–12/PA, as the PA uptake decreases, the ionic-domain sizes gradually decrease to approximately 35, 29, and 22 nm, respectively. Compared with the PA-doped ionic domains, the mechanically supportive phase formed by the PTP aromatic backbone has a higher modulus and appears as bright regions in the AFM images.^22^ These two phases are continuous and interwoven, facilitating proton conduction while maintaining membrane mechanical strength.

To further confirm the distribution of PA in the hybrid membranes, P–GSiW_11_–8/PA, which has an intermediate GSiW_11_ content, was selected as a representative sample and analyzed by AFM-based infrared spectroscopy (AFM-IR) (Fig. 3i and j). The characteristic IR absorption of the P–O bond in PA at 950 cm^−1^ was used as the imaging signal. In the AFM-IR image, the blue regions represent the mechanically supportive phase formed by the hydrophobic PTP backbone, whereas the orange regions correspond to PA-enriched ionic domains. The AFM-IR image of P–GSiW_11_–8/PA shows that PA is confined within the hydrophilic ionic domains and does not significantly affect the hydrophobic mechanically supportive phase. This spatially confined distribution helps improve PA retention and reduce the plasticization effect of PA on the polymer matrix.

### Microphase formation and PA retention mechanism

Molecular dynamics simulations were performed to reveal the formation mechanism of the microphase structure in the hybrid membranes and the distribution of PA within these membranes (Fig. 4a).^54–57^ The pristine PTP membrane exhibits a homogeneous structure, with piperidine groups uniformly distributed in the aromatic matrix (Fig. S17). After introducing GSiW_11_ into PTP, the resulting hybrid membrane shows a typical microphase-separated morphology (Fig. 4b and S18). Hybrid domains composed of GSiW_11_ and piperidine groups are formed and continuously distributed within the matrix formed by the PTP aromatic backbone, generating a three-dimensionally interconnected network-like structure.

**Fig. 4:**
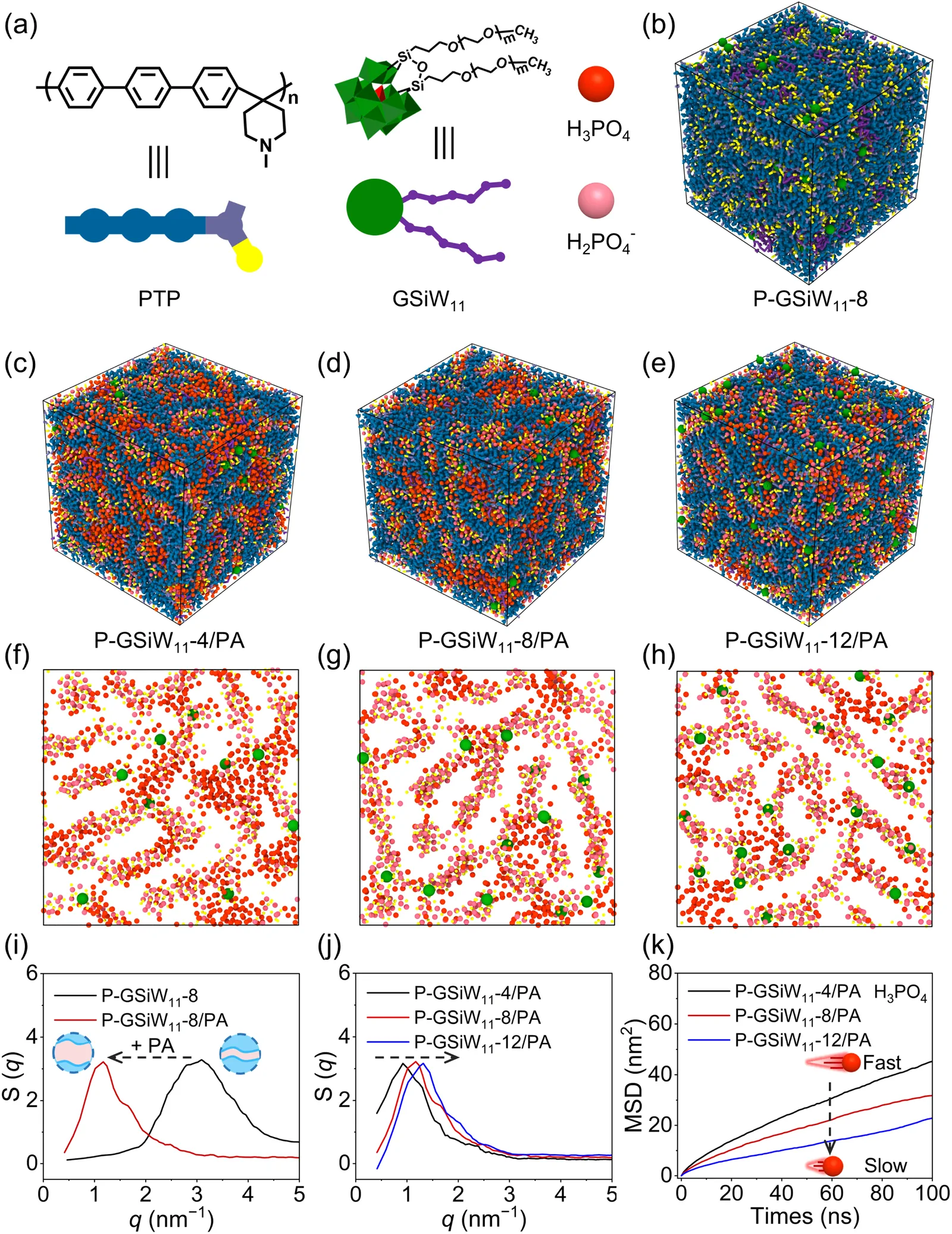
(a) Coarse-grained models of the molecules in the membranes, including PTP, GSiW_11_, H_3_PO_4_, and H_2_PO_4_^−^. Simulated snapshots of the bulk microphase structures in (b) P–GSiW_11_–8, (c) P–GSiW_11_–4/PA, (d) P–GSiW_11_–8/PA, and (e) P–GSiW_11_–12/PA. Simulated snapshots of cross-sectional hydrophilic ionic domains in (f) P–GSiW_11_–4/PA, (g) P–GSiW_11_–8/PA, and (h) P–GSiW_11_–12/PA. Structure factors of the simulated hydrophilic domains in (i) P–GSiW_11_–8 and P–GSiW_11_–8/PA and (j) P–GSiW_11_–4/PA, P–GSiW_11_–8/PA, and P–GSiW_11_–12/PA. (k) Simulated mean square displacement (MSD) of H_3_PO_4_ in P–GSiW_11_–4/PA, P–GSiW_11_–8/PA, and P–GSiW_11_–12/PA.

After PA is further introduced into the hybrid membranes, PA enters the hydrophilic ionic domains composed of GSiW_11_ and piperidine groups without disturbing the hydrophobic phase that provides mechanical support (Fig. 4c–e). The size of the hydrophilic domains increases significantly because of PA-induced swelling, while their continuity is maintained (Fig. 4f–h). Using P–GSiW_11_–8 and P–GSiW_11_–8/PA as examples, the size change of the ionic domains was further investigated using the structure factor *S*(*q*). The *S*(*q*) peak of P–GSiW_11_–8/PA shifts markedly toward lower *q* values, further supporting the enlargement of ionic domains after PA incorporation (Fig. 4i). Notably, the ionic-domain size is closely related to the PA content. The simulations show that from P–GSiW_11_–4/PA and P–GSiW_11_–8/PA to P–GSiW_11_–12/PA, the ionic-domain size gradually decreases as the PA content decreases (Fig. 4f–h), and the corresponding *S*(*q*) peak progressively shifts toward higher *q* values (Fig. 4j). These results agree well with the AFM and SAXS experiments.

To further understand how the microphase structure affects PA retention in the hybrid membranes, the mean square displacement (MSD) of PA was calculated. This method reflects the mobility of PA in the membrane, which is an important step in PA loss. From P–GSiW_11_–4/PA and P–GSiW_11_–8/PA to P–GSiW_11_–12/PA, the MSD of PA gradually decreases with increasing GSiW_11_ content (Fig. 4k), indicating that PA mobility is effectively suppressed and that PA retention is improved. This result originates from the combined effects of PA complexation by GSiW_11_ and spatial confinement of PA within the ionic domains. On the one hand, increasing the GSiW_11_ content enables denser hydrogen-bonding interactions with PA and strengthens PA binding. On the other hand, smaller ionic domains enhance the spatial confinement effect on PA. These two effects jointly improve PA retention.

### Mechanical properties and proton conductivity

PA uptake has a significant influence on the mechanical properties and proton conductivity of HT-PEMs. We investigated the saturated PA uptake of the P–GSiW_11_–*x*/PA hybrid membranes at 25 °C and compared them with a commercial PBI/PA membrane (fumapem AM-40). From P–GSiW_11_–4/PA and P–GSiW_11_–8/PA to P–GSiW_11_–12/PA, both PA uptake and swelling ratio decrease with increasing GSiW_11_ content (Fig. 5a). This is mainly because the electrostatic and hydrogen-bonding interactions between GSiW_11_ and piperidine groups occupy part of the PA adsorption sites.^22^ Notably, all hybrid membranes exhibit much lower PA uptake and swelling ratios than the PBI/PA membrane, with a more pronounced decrease in swelling ratio. The PA uptake of PBI/PA is 254%, approximately twice that of P–GSiW_11_–8/PA (125%), whereas its swelling ratio is 3.0 times higher. This is because GSiW_11_ carries multiple negative charges and can interact electrostatically with multiple piperidine groups simultaneously, thereby acting as an electrostatic cross-linker and improving the dimensional stability of the hybrid membranes.

**Fig. 5:**
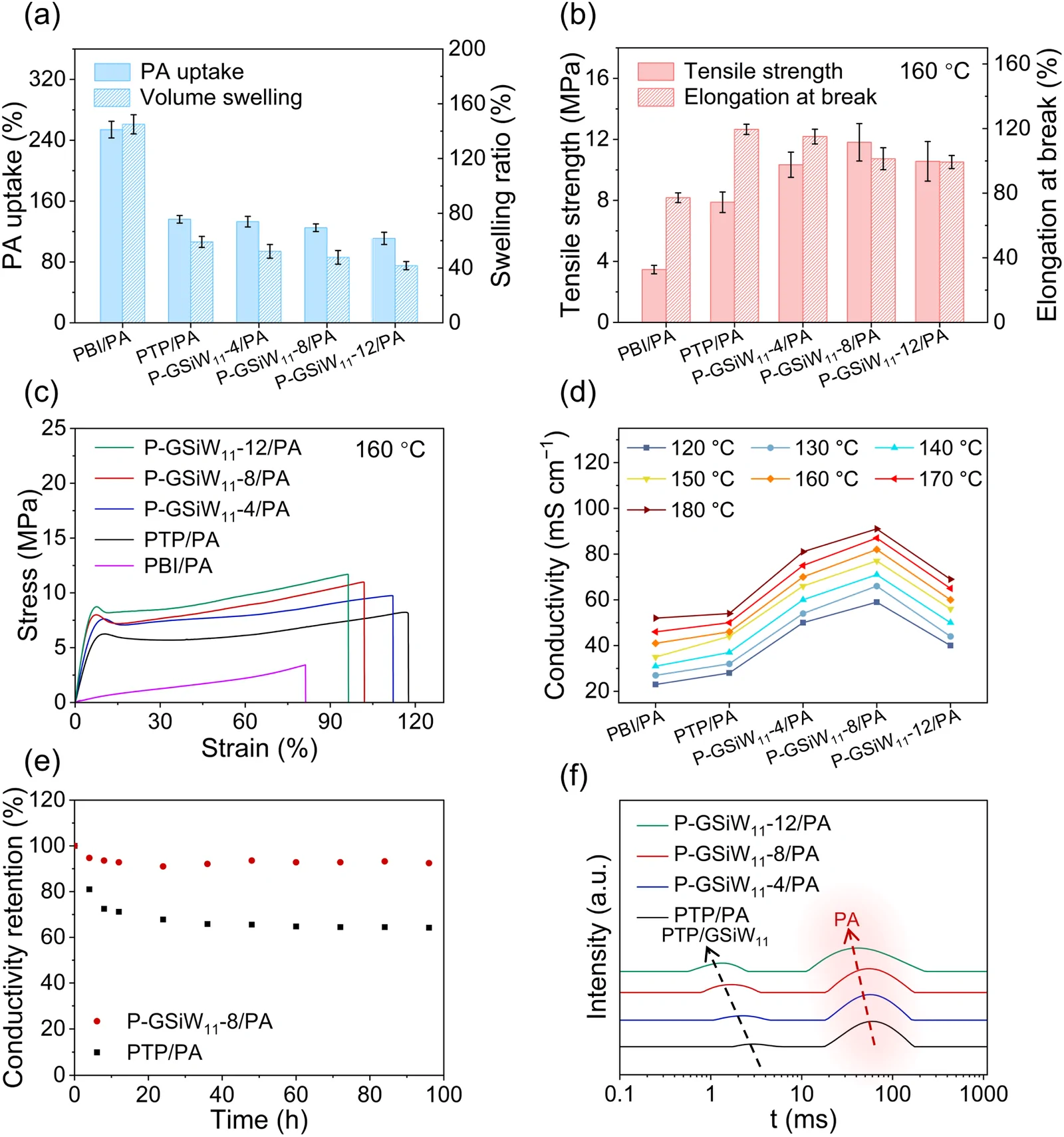
(a) PA uptake and swelling ratio of PBI/PA, PTP/PA, and P–GSiW_11_–*x*/PA membranes. (b) Mechanical properties of PBI/PA, PTP/PA, and P–GSiW_11_–*x*/PA membranes at 160 °C. (c) Stress–strain curves of PBI/PA, PTP/PA, and P–GSiW_11_–*x*/PA membranes at 160 °C. (d) Proton conductivity of PBI/PA, PTP/PA, and P–GSiW_11_–*x*/PA membranes from 120 to 180 °C under anhydrous conditions. (e) Proton-conductivity stability of PTP/PA and P–GSiW_11_–8/PA membranes at 160 °C under anhydrous conditions. (f) *T*_2_ relaxation time distributions of PTP/PA and P–GSiW_11_–*x*/PA membranes. In all cases, *x* = 4, 8, and 12.

The operating conditions of HT-PEMFCs require HT-PEMs to possess excellent mechanical properties to resist PA-induced plasticization of the membrane matrix and polymer-chain softening at high temperatures. Accordingly, we investigated the mechanical properties of these HT-PEMs. Compared with the PTP/PA membrane, the P–GSiW_11_–4/PA, P–GSiW_11_–8/PA, and P–GSiW_11_–12/PA hybrid membranes all show higher tensile strength and lower elongation at break, further confirming the electrostatic cross-linking effect of GSiW_11_ (Fig. S19). For example, the P–GSiW_11_–8/PA membrane exhibits a tensile strength of 17.5 MPa, approximately 1.3 times that of the PTP/PA membrane. Considering the high-temperature operating conditions of HT-PEMFCs, we further evaluated the mechanical properties of the membranes at 160 °C, a common operating temperature. As shown in Fig. 5b and c, the tensile strength of all membranes decreases at 160 °C, with the PBI/PA membrane showing a reduction from 18.0 to 3.4 MPa. In contrast, the hybrid membranes retain relatively high mechanical strength; in particular, P–GSiW_11_–8/PA exhibits a tensile strength of 11.8 MPa at 160 °C, approximately 3.5 times that of PBI/PA (Table 1), demonstrating its excellent high-temperature mechanical stability. The mechanical reinforcement by GSiW_11_ is attributed to three factors. First, GSiW_11_ occupies part of the PA interaction sites, reducing the saturated PA uptake of PTP and directly lowering the liquid-component content in the membrane. Second, GSiW_11_ effectively enhances interchain interactions among PTP chains through electrostatic cross-linking. Third, the microphase-separated structure of the hybrid membranes confines PA within the hydrophilic ionic domains, effectively suppressing PA plasticization of the mechanically supportive phase formed by the PTP aromatic components.^22^ These factors collectively improve the mechanical stability of the membranes and allow them to maintain high mechanical strength at elevated temperatures.

**Table 1:** Physicochemical properties of PBI/PA, PTP/PA, and P–GSiW_11_–*x*/PA hybrid membranes

Samples	PA uptake (wt%)	PA content (wt%)	Volume swelling ratio (%)	Tensile strength (MPa)^a^	Elongation at break (%)^a^	*σ* (mS cm^−1^)	
						160 °C	180 °C
PBI/PA	254 ± 11	72 ± 4	145 ± 7	3.4 ± 0.3	77 ± 3	41	52
PTP/PA	136 ± 5	59 ± 3	59 ± 4	7.9 ± 0.7	119 ± 3	46	54
P–GSiW_11_–4/PA	133 ± 7	57 ± 1	52 ± 5	10.3 ± 0.8	115 ± 5	70	81
P–GSiW_11_–8/PA	125 ± 5	55 ± 1	48 ± 5	11.8 ± 1.2	101 ± 6	82	91
P–GSiW_11_–12/PA	111 ± 8	53 ± 3	42 ± 3	10.6 ± 1.3	99 ± 4	60	69

a Measured at 160 °C.

Proton conductivity is a key parameter for evaluating HT-PEM performance. The proton conductivities of the hybrid membranes were measured under anhydrous conditions over the temperature range of 120–180 °C (Fig. 5d). The P–GSiW_11_–*x*/PA hybrid membranes show higher proton conductivities than both PTP/PA and PBI/PA membranes, indicating that introducing GSiW_11_ effectively promotes proton conduction in the hybrid membranes. This enhancement is mainly attributed to the regulation of PA proton-conducting ability and membrane microphase structure by GSiW_11_. On the one hand, GSiW_11_ forms synergistic proton conductors with PA and lowers the proton dissociation energy of PA. On the other hand, GSiW_11_ induces the self-assembly of PTP to form continuous proton-transport channels. As the GSiW_11_ content increases, the proton conductivity of P–GSiW_11_–*x*/PA first increases and then decreases. Among them, P–GSiW_11_–8/PA exhibits the highest proton conductivity, reaching 91 mS cm^−1^ at 180 °C, which is 1.7 and 1.8 times those of PTP/PA and PBI/PA membranes, respectively. Compared with P–GSiW_11_–8/PA, the proton conductivity of P–GSiW_11_–12/PA decreases to 69 mS cm^−1^. This decrease may arise from a nanocluster-crowding effect caused by the increased POM content within the ionic domains. A similar phenomenon was observed in our previously reported POM–Nafion hybrid membrane systems, where excessive POM additives reduced proton conductivity.^38,43^ The activation energies for proton conduction, derived from Arrhenius fitting, are below 0.4 eV for all membranes, corresponding to the Grotthuss-type proton conduction mechanism (Fig. S20).^22,43^ Notably, the hybrid membranes exhibit lower activation energies than the PBI/PA and PTP/PA membranes. For example, the activation energy of P–GSiW_11_–8/PA is 0.12 eV, corresponding to reductions of 43% and 29% relative to PBI/PA (0.21 eV) and PTP/PA (0.17 eV), respectively. These results indicate that the incorporation of GSiW_11_ lowers the energy barrier for proton transport in the hybrid membranes.

Because P–GSiW_11_–8/PA exhibits the highest proton conductivity, it was selected as a representative sample to investigate the long-term conductivity stability of the hybrid membrane at 160 °C without humidification (Fig. 5e). After 96 h, P–GSiW_11_–8/PA retains 92% of its initial proton conductivity, whereas the PTP/PA membrane retains only 64% under the same conditions, indicating higher PA retention in P–GSiW_11_–8/PA. To understand the mechanism of enhanced PA retention at the molecular level, LF-NMR was used to investigate PA mobility in the membranes (Fig. 5f). The PTP/PA membrane shows two peaks at 3.1 and 61.3 ms, corresponding to proton signals from the PTP matrix and PA, respectively. With increasing GSiW_11_ content, the *T*_2_ peak related to PA molecules in the P–GSiW_11_–*x*/PA membranes gradually shifts toward shorter relaxation times, indicating restricted PA molecular motion.^32,48^ These results show that introducing GSiW_11_ effectively strengthens PA immobilization in the membranes. This enhancement originates from the spatial confinement effect of nanoscale ionic domains and the complexation interactions provided by GSiW_11_ within these domains, which together reinforce the binding between PA and the membrane.

### Fuel cell performance

Because P–GSiW_11_–8/PA exhibits the highest proton conductivity, it was selected for H_2_/O_2_ fuel cell testing and compared with PTP/PA and PBI/PA membranes. At 80 °C without additional humidification, P–GSiW_11_–8/PA delivers a peak power density of 440 mW cm^−2^, twice that of the PBI/PA membrane (220 mW cm^−2^) (Fig. 6a). At 160 °C, a typical operating temperature for HT-PEMFCs, the peak power density of P–GSiW_11_–8/PA increases to 770 mW cm^−2^, 1.5 times that of PBI/PA (520 mW cm^−2^) (Fig. 6b). After applying a back pressure of 0.1/0.1 MPa to the anode and cathode to accelerate electrode reactions, the cell performance of P–GSiW_11_–8/PA is further improved, reaching a peak power density of 1130 mW cm^−2^, much higher than those of PTP/PA (870 mW cm^−2^) and PBI/PA (760 mW cm^−2^) membranes (Fig. 6c). In addition, the open-circuit voltage (OCV) of the P–GSiW_11_–8/PA membrane exceeds 0.97 V, indicating good fuel barrier properties.^20,48^

**Fig. 6:**
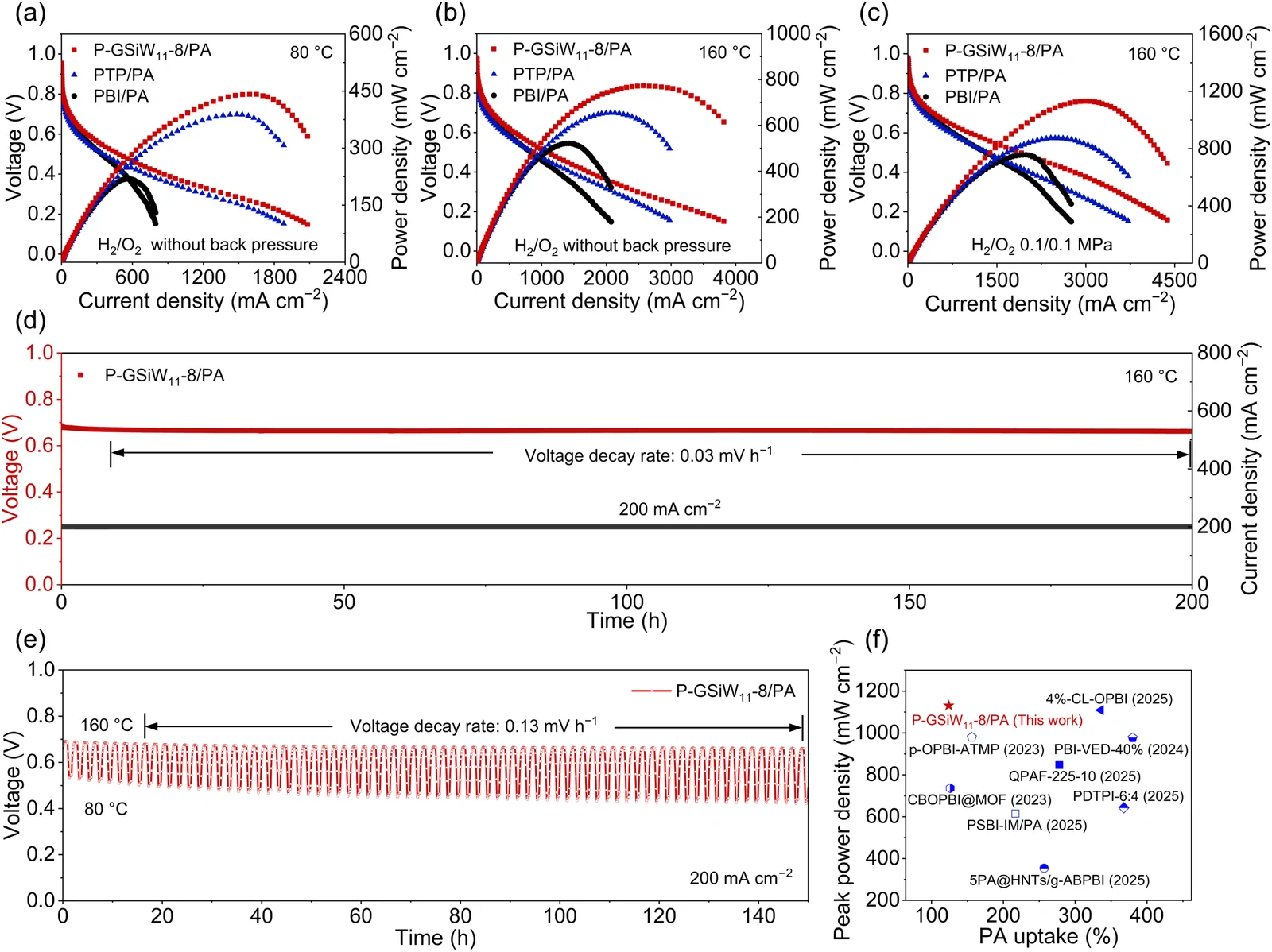
Single-cell performance based on PBI/PA, PTP/PA, and P–GSiW_11_–8/PA using dry H_2_/O_2_ at (a) 80 °C without back pressure, (b) 160 °C without back pressure, and (c) 160 °C with 0.1 MPa back pressure applied to both the anode and cathode. (d) Durability of the single cell with the P–GSiW_11_–8/PA membrane at 160 °C. (e) Thermal-cycling (80–160 °C) durability test for fuel cells based on P–GSiW_11_–8/PA membrane at 200 mA cm^−2^ without additional humidification. (f) H_2_/O_2_ fuel cell performance of the P–GSiW_11_–8/PA membrane compared with recently reported HT-PEMs. Detailed data are summarized in Table S3.

*In situ* electrochemical impedance spectroscopy (EIS) was used to evaluate the actual resistance of membrane electrode assemblies (MEAs) under operation at 0.4 V (Fig. S21). Under identical preparation conditions, the ohmic resistance (*R*_Ω_) mainly reflects the proton-transport resistance of the PEM.^58,59^ At 160 °C and 0.1/0.1 MPa, the P–GSiW_11_–8/PA-based MEA exhibits a lower *R*_Ω_ of 0.16 Ω cm^2^, outperforming PTP/PA (0.19 Ω cm^2^) and PBI/PA (0.22 Ω cm^2^), consistent with its higher proton conductivity. Meanwhile, the charge-transfer resistance (*R*_ct_) of P–GSiW_11_–8/PA is 0.06 Ω cm^2^, markedly lower than those of PTP/PA (0.15 Ω cm^2^) and PBI/PA (0.18 Ω cm^2^). This result indicates improved interfacial electrochemical reaction kinetics, which is likely associated with its higher PA retention that mitigates catalyst poisoning and interfacial proton-transport limitations caused by PA loss.^60^

The long-term stability of the single cell based on the P–GSiW_11_–8/PA membrane was further evaluated. At 80 °C and 200 mA cm^−2^, no obvious voltage decay is observed after continuous operation for 100 h, demonstrating good operational stability (Fig. S22). When the temperature is increased to 160 °C, P–GSiW_11_–8/PA shows further improved durability, with a low voltage decay rate of only 0.03 mV h^−1^ after operation at 200 mA cm^−2^ for 200 h (Fig. 6d). To further evaluate the durability of the P–GSiW_11_–8/PA-based MEA, we employed a thermal cycling protocol between 80 and 160 °C. This protocol places strict demands on the PA retention ability and dimensional stability of the PEM, as the condensed water accumulated during low-temperature operation and the repeated stress during this cycling process can both promote PA loss and compromise long-term membrane stability.^19^ The P–GSiW_11_–8/PA-based MEA maintained stable operation over 150 h throughout 75 temperature cycles, with a voltage decay rate of 0.13 mV h^−1^ at 160 °C, supporting the high PA retention and dimensional stability of the hybrid membrane (Fig. 6e). These results indicate that the P–GSiW_11_–8/PA membrane exhibits high fuel cell stability over a flexible operating temperature range of 80–160 °C. Compared with recently reported representative membranes for HT-PEMFCs, the P–GSiW_11_–8/PA membrane ranks among the top performers at low PA doping levels (Fig. 6f).

## Conclusions

In summary, this work develops a strategy that integrates supramolecular complexation with nanoconfinement to achieve efficient and stable proton conduction in PA-doped polymer membranes. A self-assembled GSiW_11_ additive was designed and introduced into a PTP matrix. Through supramolecular complexation between GSiW_11_ and PA, a synergistic proton conductor is formed that promotes PA proton dissociation. In addition, GSiW_11_ co-assembles with PTP to form nanoscale and continuous ionic domains, where PA is immobilized to construct efficient and stable proton-conducting networks. Based on these effects, hybrid proton exchange membranes with high proton conductivity and conduction stability were prepared. The optimized membrane achieves excellent proton conductivity (91 mS cm^−1^ at 180 °C), high mechanical strength (11.8 MPa at 160 °C), and stable PA retention at a relatively low PA uptake of 125%. The corresponding single cell delivers a peak power density above 1100 mW cm^−2^ at 160 °C and exhibits good operational stability over the temperature range of 80–160 °C. This work demonstrates the unique advantages of self-assembled POMs in enabling PA immobilization and facilitating PA-mediated proton conduction, providing a new design strategy for high-performance HT-PEMs with broad operating temperature windows.

## Author contributions

Tingting Li: conceptualization, data curation, formal analysis, methodology, visualization, software, writing – original draft. Yi Zhang: software, visualization, methodology. Peng Zuo: visualization, formal analysis. Shihao Song: visualization, formal analysis. Rong-Lin Zhong: software, formal analysis, methodology, visualization. Haolong Li: supervision, conceptualization, formal analysis, funding acquisition, resources, writing – review & editing.

## Conflicts of interest

There are no conflicts to declare.

## Supplementary Material

SC-017-D6SC05807H-s001

## Data Availability

The data supporting this article have been included as part of the supplementary information (SI). Supplementary information: experimental procedures, DFT calculations, NMR spectra, TGA curves, DSC curves, *etc.* See DOI: https://doi.org/10.1039/d6sc05807h.
