# Supplementary material for: Self-assembled polyoxometalate enables efficient and stable proton conduction in high-temperature polymer electrolyte membranes
Source: Chem Sci. 2026 Aug 26;17(40):19198–208. doi: 10.1039/d6sc05807h (PMC13536948; doi:10.1039/d6sc05807h)
Supplement: Supplementary file 1 [file SC-017-D6SC05807H-s001.pdf]

Supplementary Information (SI) for Chemical Science.
This journal is © The Royal Society of Chemistry 2026
Supplementary Information (SI) for Chemical Science.
This journal is © The Royal Society of Chemistry 2026

Supporting Information

Self-Assembled Polyoxometalate Enables Efficient and Stable Proton Conduction in High-Temperature Polymer Electrolyte Membranes

Tingting Li, Yi Zhang, Peng Zuo, Shihao Song, Rong-Lin Zhong and Haolong Li*

State key laboratory of supramolecular structure and materials, College of Chemistry, Jilin University, Changchun 130012, China

*Corresponding author. Email: hl_li@jlu.edu.cn

Contents

1. Materials S1
2. Synthesis S1
   2.1 Synthesis of PTP polymer S1
   2.2 Synthesis of polyethylene glycol silane [$CH_3(OCH_2CH_2)_nO(CH_2)_3Si(OCH_3)_3$] S1
   2.3 Synthesis of $H_4[SiW_{11}O_{39}(SiC_{2n+4}H_{4n+9}O_{n+1})_2O]$ ($GSiW_{11}$) S1
3. Hybrid membranes preparation S2
   3.1 Preparation of binary hybrid membranes S2
   3.2 PA uptake and volume swelling S2
4. Characterization S2
5. Simulation details S4
6. Supplementary figures S6
7. Supplementary tables S15
8. References S16

1. Materials

Trifluoroacetic acid (TFA, 99%), trifluoromethanesulfonic acid (TFSA, 98%), p-terphenyl, and N-methyl-4-piperidone (99%) were purchased from Aladdin. Polyethylene glycol monomethyl ether [$CH_3(OCH_2CH_2)_nOH$] (mPEG, M_w = 500 g mol^{-1}), tetrahydrofuran (THF, 99.9%, extra dry, with molecular sieves), (3-chloropropyl)trimethoxysilane, and sodium hydride (NaH, 60% dispersion in mineral oil) were purchased from Energy Chemical. Phosphoric acid (PA, 85 wt%), potassium carbonate, ethanol, dichloromethane, thionyl chloride (99%), and N-methyl-2-pyrrolidone (NMP) were provided by Beijing Chemical Works. $H_4SiW_{12}O_{40}$ (SiW_{12}), Sodium tungstate dihydrate and sodium silicate were bought from Sigma-Aldrich without additional purification. $K_8[SiW_{11}O_{39}]$ was prepared by following a procedure from the literature.[S1] PBI membrane (Fumapem AM-40) was purchased from SCI Materials Hub.

2. Synthesis

2.1 Synthesis of PTP polymer

PTP polymer was synthesized by superacid-catalyzed polymerization.[S2] p-terphenyl (5.0 g, 1 equiv.) was dispersed in 13.0 mL of CH_2Cl_2, followed by the addition of N-methyl-4-piperidone (3.2 mL, 1.3 equiv.). Then TFA (1.6 mL) and TFSA (19.0 mL) were sequentially added dropwise in an ice bath. After stirring for 5-6 h, the resulting dark-blue viscous mixture was poured into methanol, yielding a light-yellow fibrous polymer. The product was washed twice with deionized water and subsequently with methanol, followed by drying in a vacuum oven at 60 °C for 24 h.

2.2 Synthesis of polyethylene glycol silane [$CH_3(OCH_2CH_2)_nO(CH_2)_3Si(OCH_3)_3$]

mPEG (3.0 mL, 1.0 eq.) was dissolved in 20 mL of tetrahydrofuran (THF). Then NaH (0.30 g, 1.2 eq.) was added slowly with stirring. After the evolution of hydrogen bubbles ceased, the reaction system was purged with nitrogen for 30 min to remove air. Subsequently, (3-chloropropyl)trimethoxysilane (2.4 mL, 2.0 equiv.) was added, and the mixture was stirred at 40 °C for 8 h. The reaction mixture was centrifuged to remove NaCl and concentrated by rotary evaporation. The concentrated product was slowly added into n-hexane, yielding a light-yellow transparent liquid after separation.

2.3 Synthesis of $H_4[SiW_{11}O_{39}(SiC_{2n+4}H_{4n+9}O_{n+1})_2O]$ ($GSiW_{11}$)

$GSiW_{11}$ was synthesized according to the method previously reported by our group.[S3, S4] $K_8[SiW_{11}O_{39}]$ (1.0 eq.) was dispersed in 20 mL of acetonitrile (MeCN), followed by the sequential addition of polyethylene glycol silane (2.5 eq.) and 1.0 M HCl (5.0 eq.). After stirring overnight at room temperature, the mixture was filtered and the solvent was removed by rotary evaporation. The residual solid was dissolved in dimethylformamide (DMF) to obtain a light-yellow solution, which was then filtered and slowly added to chloroform to obtain a light-yellow solid. This step was repeated 2–3 times to obtain $K_4[SiW_{11}O_{39}(SiC_{2n+4}H_{4n+9}O_{n+1})_2O]$. To replace the K^+ counterions with H^+, the $K_4[SiW_{11}O_{39}(SiC_{2n+4}H_{4n+9}O_{n+1})_2O]$ was dissolved in DMF and slowly added dropwise into 0.5 M HCl. The mixture was maintained at 60 °C for 12 h to facilitate ion exchange. After ion exchange, the HCl solution was decanted, and the product was repeatedly washed with deionized water until the filtrate became neutral. Finally, the product was dried at 60 °C for 24 h to obtain $H_4[SiW_{11}O_{39}(SiC_{2n+4}H_{4n+9}O_{n+1})_2O]$. Elemental analysis (EA) of $GSiW_{11}$ complex (%): Calculated for $K_4[SiW_{11}O_{39}(SiC_{2n+4}H_{4n+9}O_{n+1})_2O]$ (n = 10, M_w = 3930 g mol^{-1}): C: 14.67%, H: 2.51%. Found: C: 14.55%, H: 2.66%. Inductively coupled plasma (ICP) analysis of $GSiW_{11}$ gave the following results: ρK (mg L^{-1}) = 0.446, ρW (mg L^{-1}) = 98.7; Ratio: K/W = 0.229/11, indicating that K^+

counterions were almost completely replaced.

3. Hybrid membranes preparation

3.1 Preparation of binary hybrid membranes

The PTP polymer was dissolved in NMP at room temperature, followed by the addition of a few drops of trifluoroacetic acid. Subsequently, a $GSiW_{11}$/NMP solution (20 mg mL^{-1}) was slowly added into the PTP/NMP solution under stirring, with the $GSiW_{11}$ content varying from 0 wt% to 12 wt%. The resulting mixtures were stirred at room temperature for 6 h and then cast onto glass substrates. The solvent was evaporated in an oven at 70 °C for 24 h. Finally, the obtained membranes were peeled from the substrates and further dried in a vacuum oven at 120 °C for 24 h. The resulting hybrid membranes were denoted as P-$GSiW_{11}$-x, where x (x = 0, 4, 8, and 12) represents the weight percentage of $GSiW_{11}$ relative to the total weight of $GSiW_{11}$ and pristine PTP, that is

$$x = \frac{m_{GSiW}}{m_{GSiW} + m_{PTP}} \times 100\%$$

3.2 PA uptake and volume swelling

The PTP and P-$GSiW_{11}$-x membranes were immersed in 85 wt% PA at 25 °C for 24 h to reach equilibrium absorption, whereas the PBI membranes were immersed in 85 wt% PA at 140 °C for 24 h to achieve the PA uptake recommended by the manufacturer. Subsequently, the excess PA on the membrane surfaces was carefully removed using filter paper, followed by drying in a vacuum oven at 120 °C for 12 h. The obtained PA-doped membranes were denoted as PTP/PA, P-$GSiW_{11}$-x/PA, and PBI/PA, respectively. The weights and volumes of the membrane samples were measured before and after PA doping. The PA uptake, PA content, and volume swelling ratio were calculated based on the average values obtained from different membrane samples. The corresponding values were determined according to the following equations.

$$PA\ uptake\ (\%) = \frac{W_u - W_d}{W_d} \times 100\% \qquad (1)$$

$$PA\ content\ (\%) = \frac{W_u - W_d}{W_u} \times 100\% \qquad (2)$$

$$Volume\ swelling\ (\%) = \frac{V_u - V_d}{V_d} \times 100\% \qquad (3)$$

where W_d and W_u represent the weights of the membranes before and after PA doping, respectively, while V_d and V_u represent the volumes of the membranes before and after PA doping, respectively.

4. Characterization

^1H NMR spectra were recorded on a Bruker AVANCE III 500 MHz spectrometer at room temperature. Elemental analysis (EA) was performed using a Vario Micro Cube elemental analyzer (AGILENT America). Inductively coupled plasma spectrometry (ICP) measurements were conducted using an Agilent 725 instrument, with samples dissolved in 5 wt% nitric acid solution prior to analysis. Attenuated total reflection-Fourier transform infrared (ATR-FTIR) spectra of the membranes were measured on a Thermo Scientific™ Nicolet™ iS™10 FTIR in ATR mode at a resolution of 4 cm^{-1} in the range of 4000–400 cm^{-1}. Thermogravimetric analysis (TGA) was carried out on a Mettler Toledo TGA2 under a nitrogen atmosphere at a heating rate of 10 °C min^{-1} from 80 to 800 °C. Differential scanning calorimetry (DSC) measurements were performed on a TA Instruments DSC Q20 at a heating rate of 10 °C min^{-1}, and all reported data were obtained from the

second heating cycle. Low-field ^{1}H NMR (LF-NMR) was performed using a Niumag MesoMR23-040V low-field spectrometer at 20 MHz proton resonance frequency. X-ray diffraction (XRD) patterns were recorded on an Empyrean X-ray diffractometer (PANalytical B.V.), using Cu $K_{\alpha 1}$ radiation of a wavelength of 1.542 Å. Small-angle X-ray scattering (SAXS) measurements of the undoped membranes were performed on Xenocs 3.0 instrument with an X-ray wavelength of 1.542 Å. The distance between the sample and the detector was 200 mm, and the membranes were exposed to the X-ray source for 6 min. SAXS measurements of the PA-doped membranes were conducted using an Anton Paar SAXSess mc2 instrument with an X-ray wavelength of 1.542 Å. Transmission electron microscopy (TEM) images were obtained using a JEM-2100F electron microscope operated at an accelerating voltage of 200 kV. Ultrathin sections (~80 nm) of the PTP and hybrid membranes were prepared for TEM characterization. Atomic force microscopy (AFM) images were recorded on a Bruker ICON-XR microscope in tapping mode under ambient conditions. AFM-IR images were acquired by scanning the AFM tip across the sample surface at 950 cm^{-1} in tapping mode. The tensile strength and elongation at break of the membranes were evaluated using a universal testing machine (Instron 68TM-5) at room temperature and 160 °C with a stretching rate of 2.0 mm min^{-1}.

Proton Conductivity. The proton conductivity (σ) of the HT-PEMs was measured on a Princeton Applied Research Model 2273 electrochemical workstation using the four-probe method over the frequency range of 100 kHz to 0.01 Hz in the temperature range of 120–180 °C under anhydrous conditions. The dimensions of the PEMs were approximately 1 cm × 4 cm, and the AC perturbation voltage was set to 10 mV. σ was calculated using the following equation:

$$\sigma = \frac{L}{RS}$$

where L and S denote the distance between the two electrodes and the cross-sectional area of the membrane, respectively, and R denotes the resistance of the membrane.

Conductivity stability. The HT-PEMs were maintained at 160 °C under 0% RH. The proton conductivity was measured at different time intervals, and the proton conductivity retention was calculated according to the following equation.

$$Conductivity\ retention\ (\%) = \frac{\sigma_t}{\sigma_i} \times 100\%$$

where σ_i denotes the initial proton conductivity and σ_t denotes the proton conductivity after a period of time.

Single-cell performance. MEAs for fuel cell testing were prepared using the catalyst-coated substrate method. The catalyst ink was prepared by mixing Pt/C catalyst and PTFE emulsion with water, followed by the addition of isopropyl alcohol (water/isopropyl alcohol = 1:7 by weight). The resulting mixture was ultrasonicated for 60 min to obtain a homogeneous catalyst ink. The obtained catalyst ink was then coated onto carbon paper with a gas diffusion layer to prepare the gas diffusion electrodes (GDEs). The Pt loading was 1.0 mg cm^{-2}, and the mass ratio of catalyst to PTFE binder was 8:2. The MEAs were fabricated by sandwiching the membrane between two GDEs with an active area of 5 cm^2. Dry H_2 and O_2 were supplied to the anode and cathode at flow rates of 200 mL min^{-1}, respectively. Single-cell polarization measurements were performed at 80 and 160 °C without back pressure. Additional measurements at 160 °C were conducted with a back pressure of 100 kPa applied to both the anode and cathode. Prior to testing, the MEAs were activated at a constant current

density until a stable cell voltage was reached. Single-cell stability tests were carried out at a constant current density of 0.2 A cm^{-2} using H_2/O_2 flow rates of 200/200 mL min^{-1} without back pressure. The single-cell thermal-cycling durability test was conducted at a constant current density of 200 mA cm^{-2}, with H_2 and O_2 flow rates of 200 mL min^{-1} and no back pressure. Each temperature cycle lasted 2 h, and the cell was continuously operated throughout both the heating and cooling stages. Electrochemical impedance spectroscopy (EIS) measurements were performed on a VersaSTAT3 electrochemical workstation at 160 °C and 0.4 V over the frequency range from 10 kHz to 0.01 Hz, with a back pressure of 100 kPa applied to both the anode and cathode. The H_2/O_2 flow rates were the same as those used for the polarization measurements.

5. Simulation details

Molecular dynamics (MD) simulations were performed using a coarse-grained (CG) model to investigate the formation of the microphase-separated structure of the hybrid membrane and the distribution of PA within the membrane. The CG models of PTP and $GSiW_{11}$ were constructed based on the Martini force field.[S5, S6] The reduced units for length, energy and mass are σ_0 = 1.0 nm, ϵ_0 = 1.0 kJ mol^{-1}, and m_0 = 1.0 amu, respectively. The non-bonded interactions among the CG beads were described using the Lennard–Jones (LJ) potential:

$$U_{\mathrm{LJ}}(r) = 4.0\epsilon\left[\left(\frac{\sigma}{r}\right)^{12} - \left(\frac{\sigma}{r}\right)^{6}\right]$$

where r is the distance between two adjacent beads, and r_c = 1.8 σ_0 is the cutoff distance. The interaction parameter ϵ ranges from 1.8ε_0 to 5.6ε_0 according to the Martini force field. The parameter σ, representing the effective bead size, is set to 1.0 σ_0 for $GSiW_{11}$ beads and 0.47 σ_0 for all other CG beads.

The electrostatic interactions between charged beads are described by Coulomb potential:

$$U_{Coul} = \frac{q_i q_j \varepsilon}{4\pi\varepsilon_0\varepsilon_r r}$$

where q_i and q_j denote the charges of beads i and j, respectively, separated by a distance r. ε denotes the vacuum permittivity, and the dielectric constant is set to ε_0=1.0 that is consistent with the original model.[S7] The charges of $GSiW_{11}$ and the dihydrogen phosphate are set to −4 and −1, respectively. To maintain charge neutrality of the system, the charges of the hydrophilic beads of the piperidinium groups are set to +1 or 0 depending on the total negative charge in the system. Specifically, in the PA-doped systems, all hydrophilic beads of the piperidinium groups are assigned a charge of +1. In contrast, for the systems without PA, the number of positively charged hydrophilic beads of the piperidinium groups is determined according to the amount of $GSiW_{11}$ present in the system. The Coulomb interactions are calculated by the particle-particle particle-mesh method with an accuracy of 10^{-5}.[S8]

Harmonic potential is employed to describe bond stretching interactions:

$$U_{bond}(r) = \frac{1}{2}K_{bond}(r - r_0)^2$$

where r_0 = 0.47 σ_0 and K_{bond} = 1250 ϵ_0/σ_0^2. The angle bending interactions for PTP chains are described by the harmonic potential:

$$V_{\mathrm{angle}}(r) = \frac{1}{2}K_{\mathrm{angle}}[cos(\theta) - cos(\theta_0)]^2$$

where K_{angle} = 25 ϵ_0 and θ_0 = 180 or 110 degrees.

The structure factor S(q) is calculated using the following equation:

$$S(q) = \rho_A(q)\rho_A(-q)/N_A$$

$$\rho(q) = \sum_{i=1}^{N} exp\,(iq\,.\,r)$$

where N_A denotes the total number of A beads in the reciprocal space, and ρ(q) represents the spatial Fourier transform of the number density.

Under an electric field, the dynamic behavior of charged beads is characterized by the mean-square displacement (MSD), which is calculated using the following equation:

$$MSD(t) = \frac{1}{M}\sum_{i=1}^{M}[\,(r_i(t) - r_i(0)]^2$$

where r_i(t) denotes the position vector of beads i at time t.

The simulation system consists of 100 coarse-grained PTP chains, with each chain containing 50 monomers and 50 hydrophilic beads of the piperidinium groups. For the P-GSiW_{11}-4, P-GSiW_{11}-8, and P-GSiW_{11}-12 hybrid membranes, 76, 103, and 163 GSiW_{11} beads are additionally introduced into the simulation box, respectively, according to the mass fraction of GSiW_{11} in the hybrid membranes. Considering that the PA uptake of the hybrid membranes gradually decreases with increasing GSiW_{11} content in the experiments, the PA contents in the simulated systems are adjusted following the same trend. Accordingly, based on the relative proportions of PA uptake in the hybrid membranes, the PA contents in the P-GSiW_{11}-4/PA, P-GSiW_{11}-8/PA, and P-GSiW_{11}-12/PA systems are set to 38%, 35%, and 32%, respectively. The numbers of phosphoric acid beads and dihydrogen phosphate beads are determined according to the prescribed PA contents and the charge neutrality condition of the systems. The molecules are initially randomly placed in the simulation box. The simulations are performed in a cubic box with three-dimensional periodic boundary conditions in the isothermal-isobaric ensemble. The equilibrium configurations are obtained by an annealing cycle simulation with a total duration of 80 ns, during which the temperature is linearly increased from 298 K to 894 K and subsequently decreased back to 298 K. The beads are integrated forward in time according to the Newton's equations of motion with a time step dt = 0.002 ps. The simulation temperature is maintained at 298 K using a Nosé–Hoover thermostat.[S9, S10]

Density functional theory (DFT). All DFT calculations were performed using the Gaussian 16 program.[S11] The B3LYP functional[S12-S15] with Grimme's semiempirical dispersion potential D3(BJ) was used for geometry optimization.[S16] The geometry optimizations were performed using the B3LYP functional in the gas phase and the 6–31G(d) basis sets. For the basis sets, W atom was described using the LANL2DZ basis sets[S17] and the 6-31G(d) basis set was used for all other atoms. Single-point energy calculations were performed with the B3LYP-D3(BJ) functional and a mixed basis set of SDD[S18] for W and 6-311++G(d,p) for the other atoms. Solvent effects were considered by the continuum solvation model SMD developed by Truhlar's group.[S19]

The proton affinities were calculated in the gas phase using the following equation:

$$\text{Proton affinity} = E(\text{HA}) - E(A^-) - E(\text{H}^+)$$

where E(AH) and E(A^-) denote the calculated energies of the AH and its conjugate base A^-, respectively. The proton energy was evaluated using a hydrated-proton reference rather than an isolated H^+.

6. Supplementary Figures

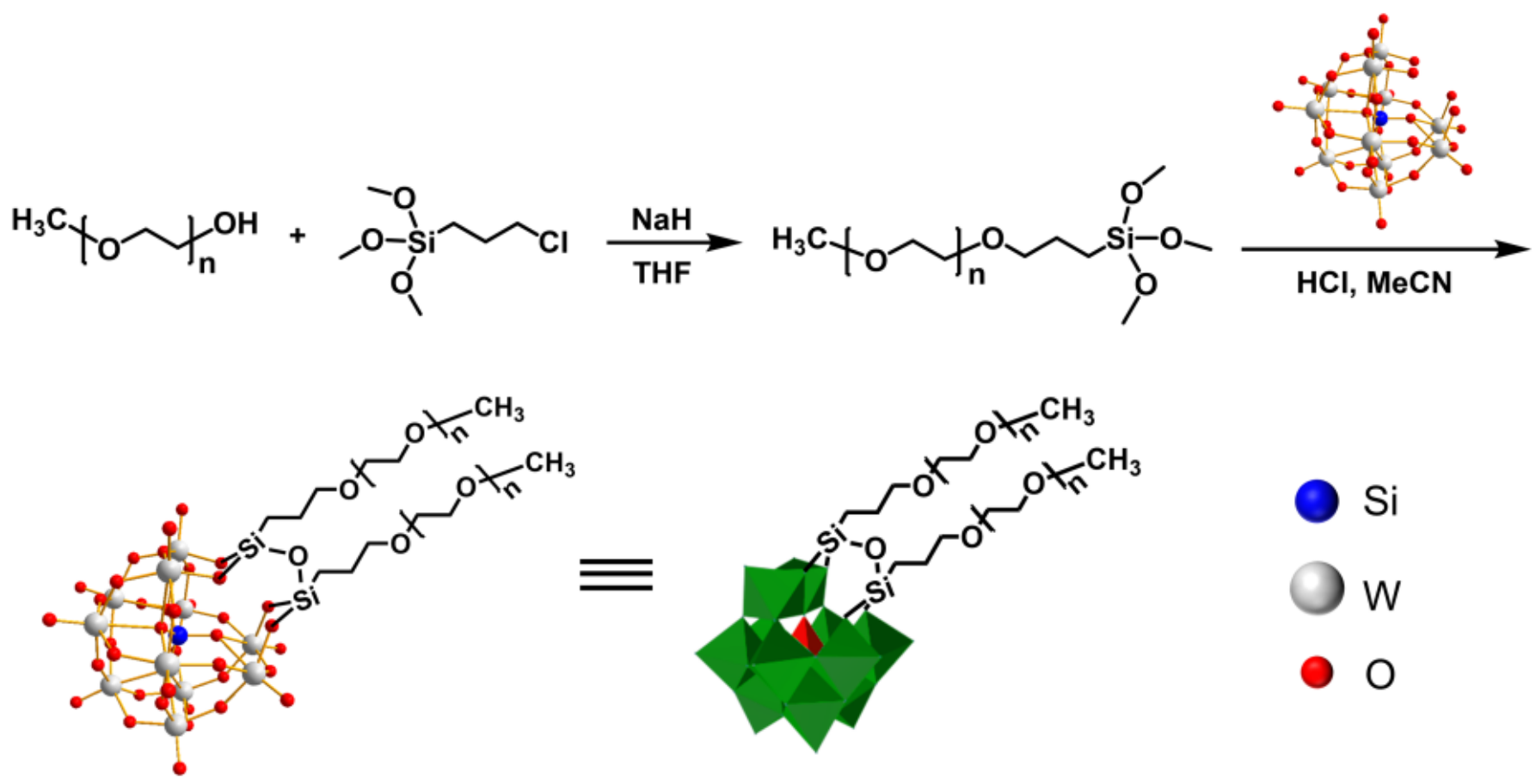

Scheme S1. Synthesis route of $GSiW_{11}$.

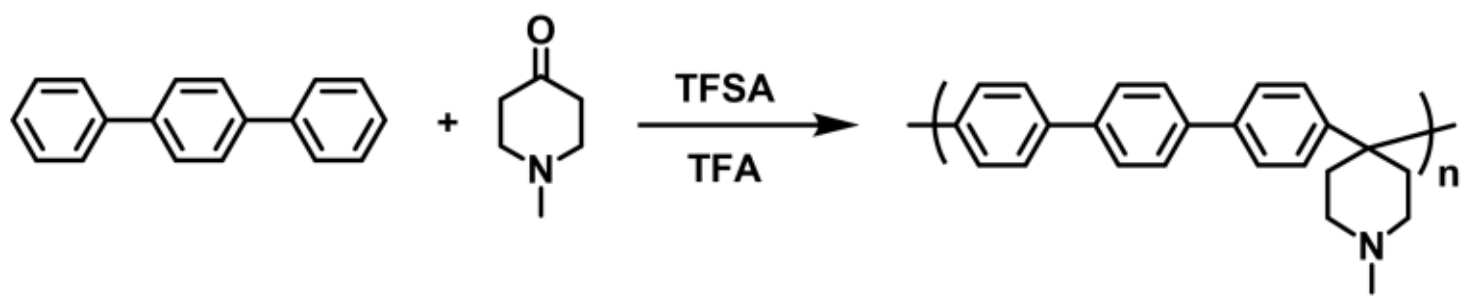

Scheme S2. Synthesis route of PTP.

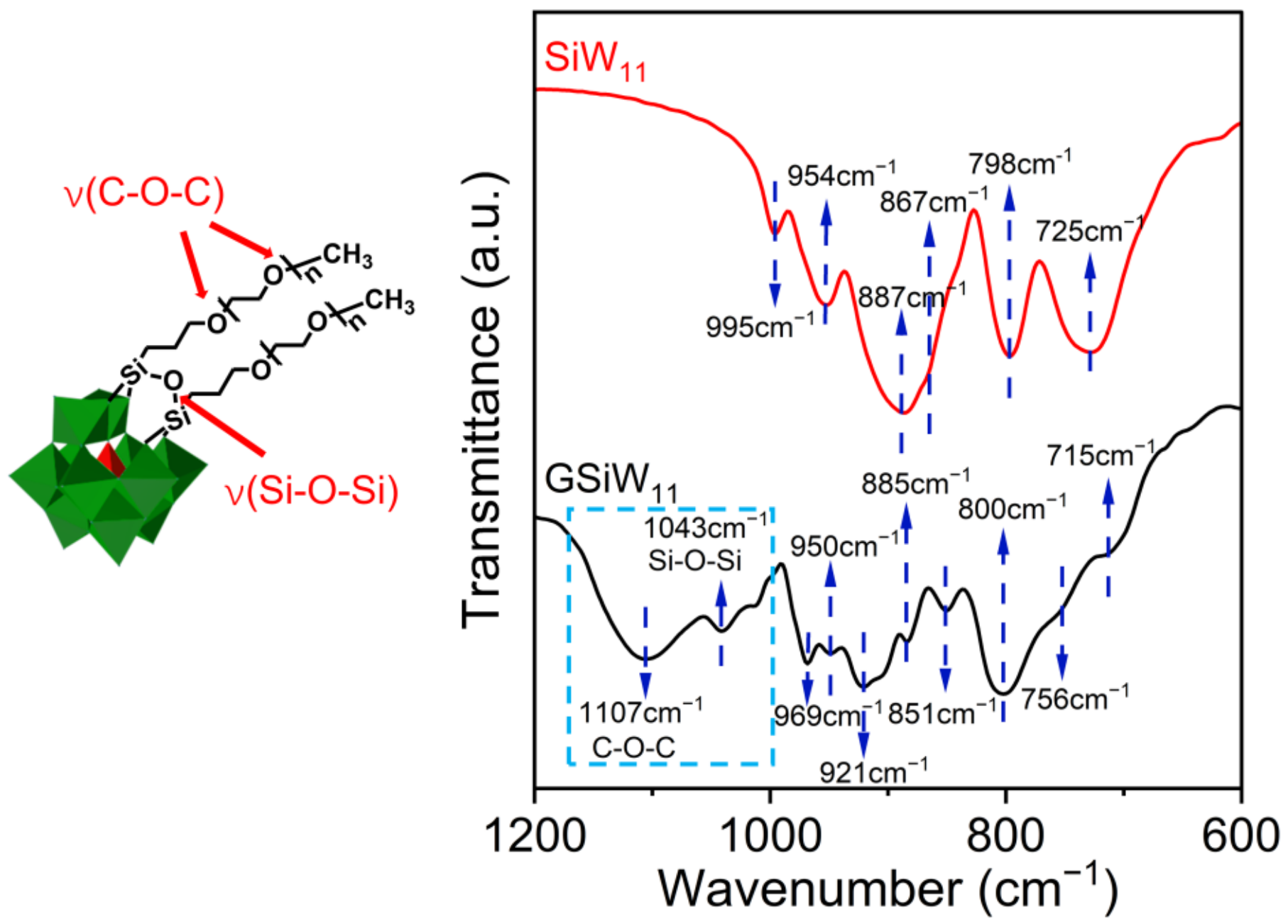

Figure S1. FTIR spectra of SiW_{11} and $GSiW_{11}$. Four kinds of oxygen atoms in pure SiW_{11} give rise to characteristic vibration bands at 725 cm^{-1} and 798 cm^{-1} (W–O_c–W), 867 cm^{-1} (W–O_b–W), 887 cm^{-1} (Si–O_a), 954 cm^{-1} and 995 cm^{-1} (W=O_d), respectively (O_c, edge-sharing oxygen; O_b, corner-sharing oxygen; O_d, terminal oxygen atom to W; O_a oxygen in SiO_4 tetrahedron). Obvious shifts of the absorption peaks are observed corresponding to the vibration of W–O_c–W at 715 cm^{-1}, 756 cm^{-1} and 800 cm^{-1}, W–O_b–W at 851 cm^{-1}, Si–O_a at 885 cm^{-1}, W=O_d at 921 cm^{-1}, 950 cm^{-1} and 969 cm^{-1} in $GSiW_{11}$.

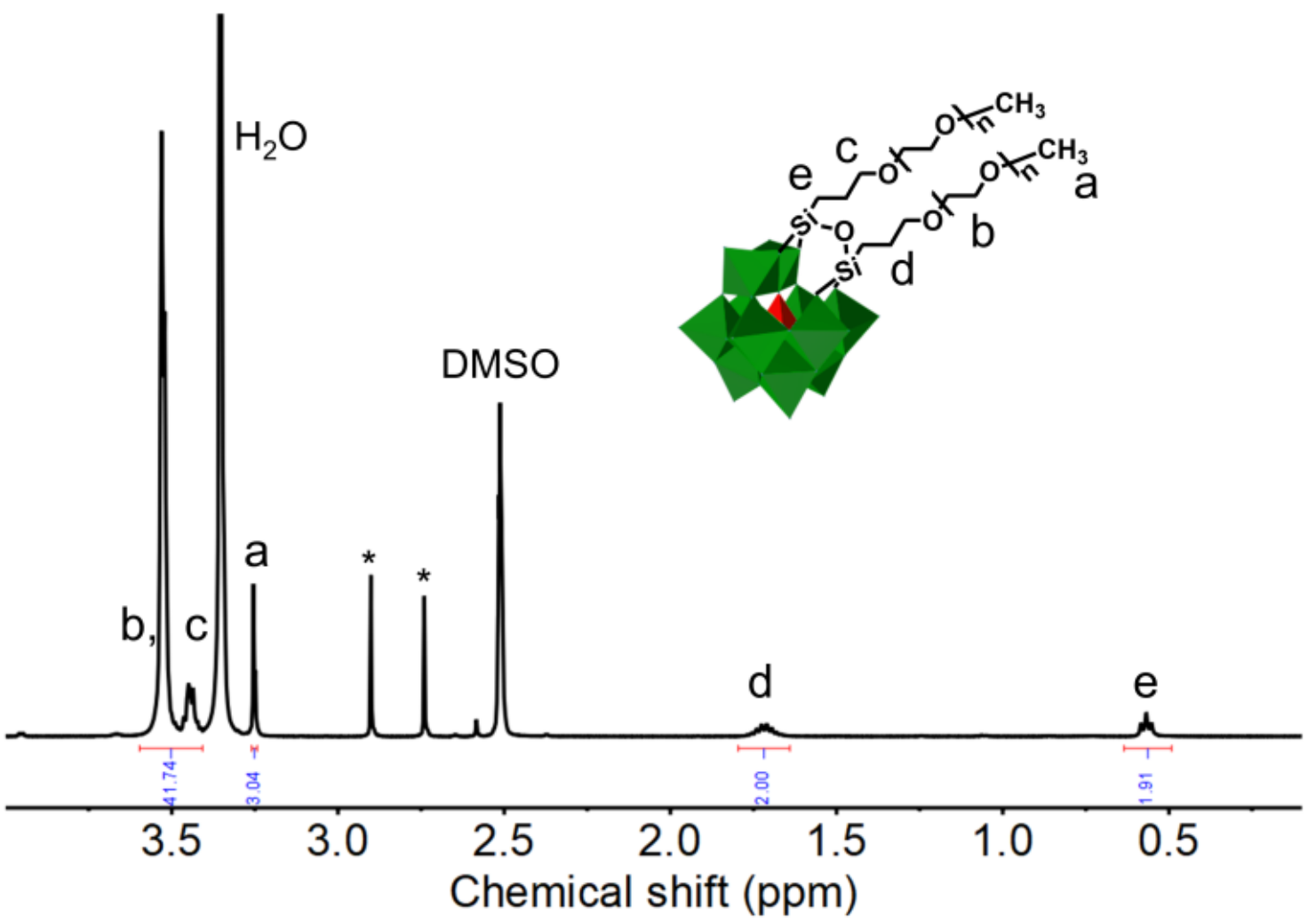

Figure S2. ^{1}H NMR spectrum of $GSiW_{11}$ in DMSO-d_6.

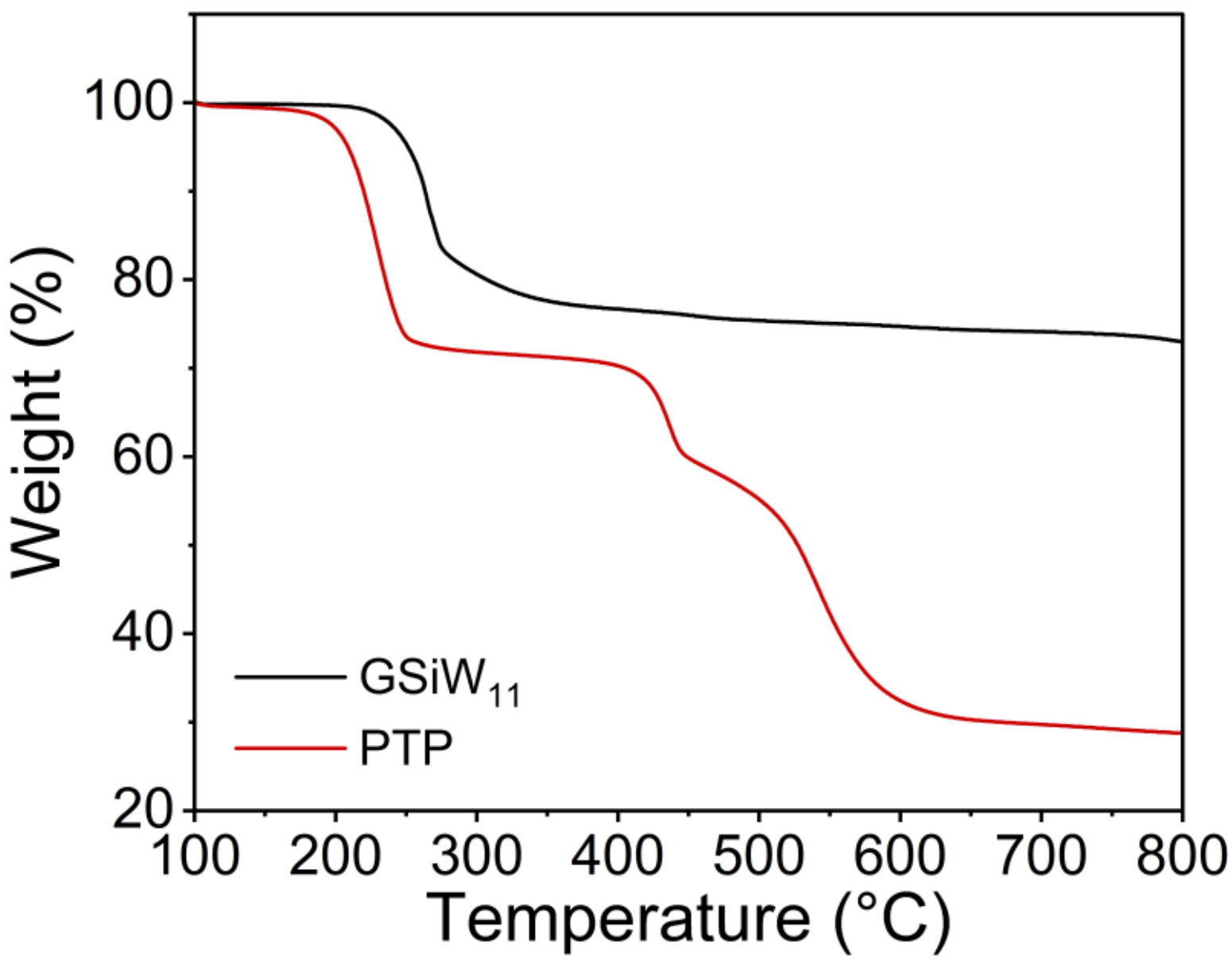

Figure S3. TGA curves of $GSiW_{11}$ and PTP.

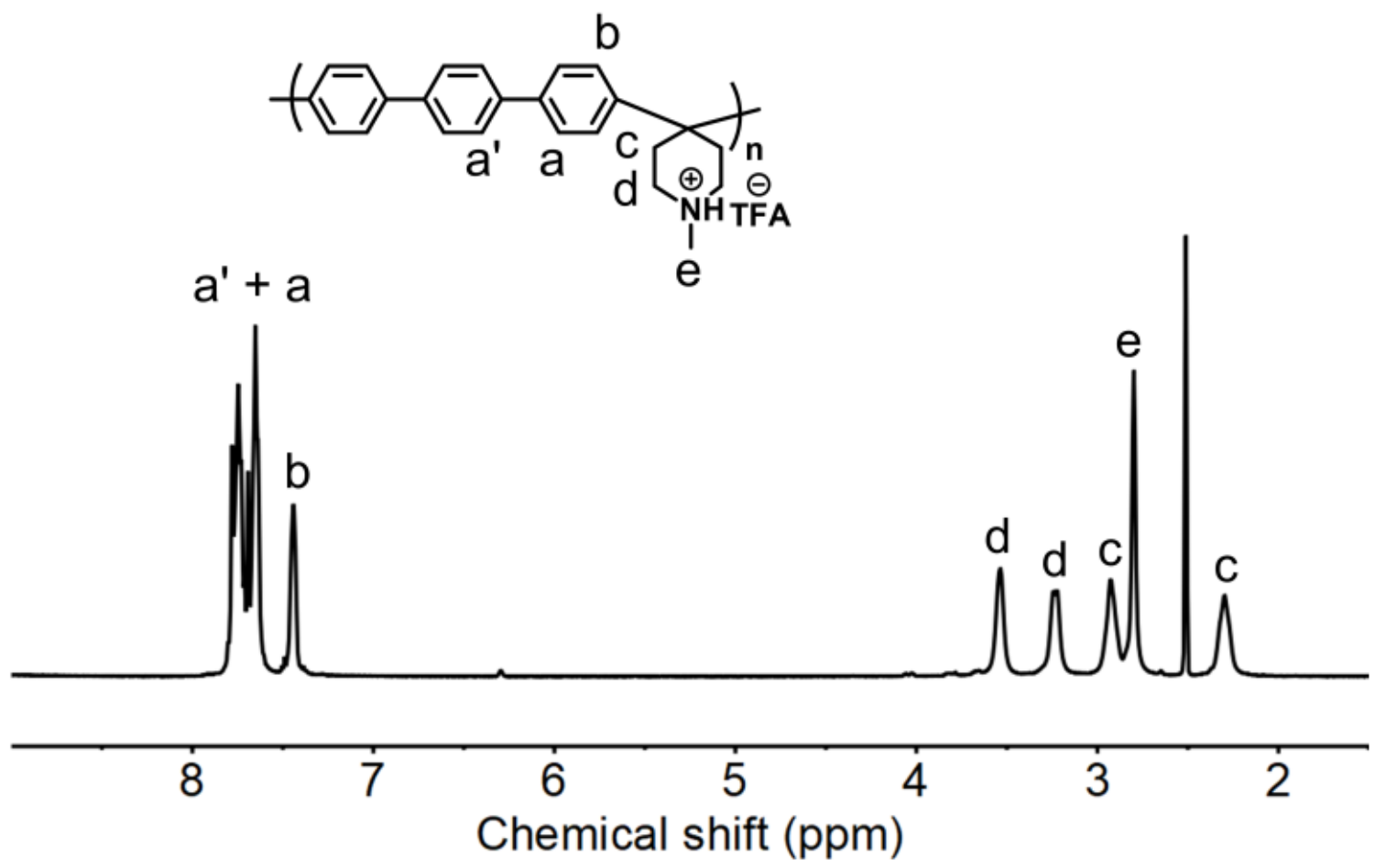

Figure S4. ^{1}H NMR spectrum of PTP in DMSO-d_6 containing 3–6 vol% of TFA.

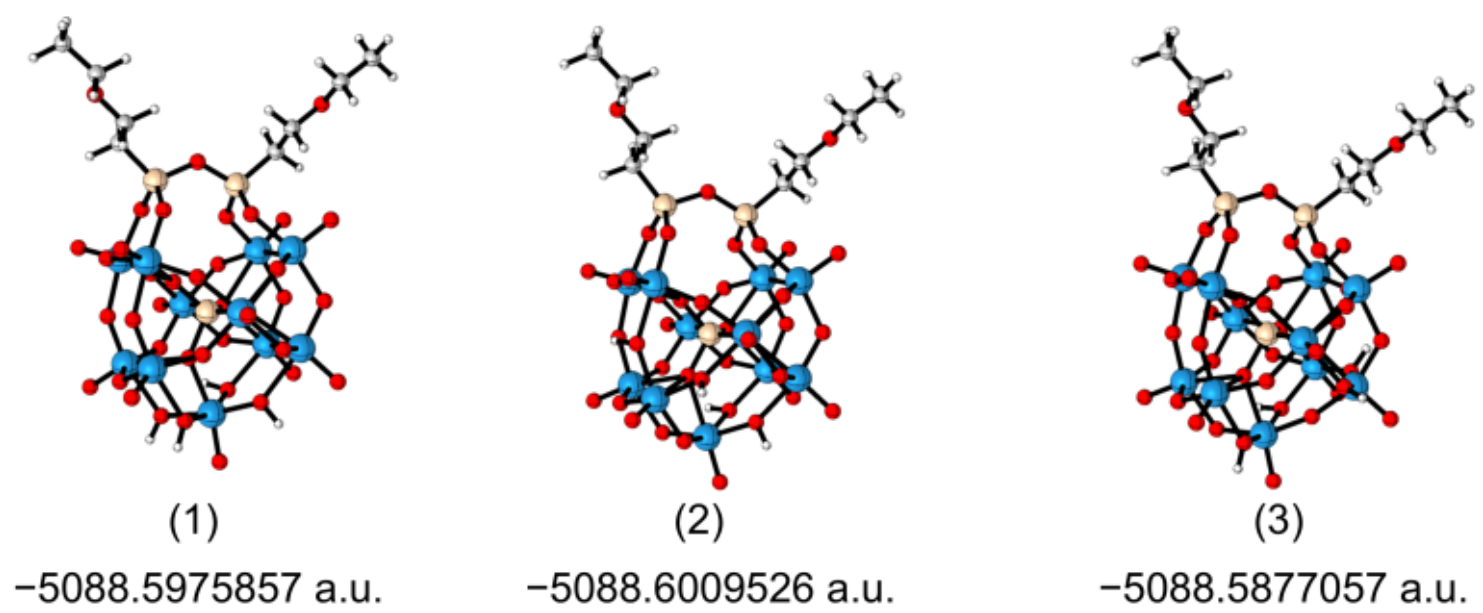

Location	1	2	3
ΔG (kcal mol^{-1})	2.11	0.00	8.31

Figure S5. Among the four H^+ ions of $GSiW_{11}$ at different positions, position 2 is the most stable.

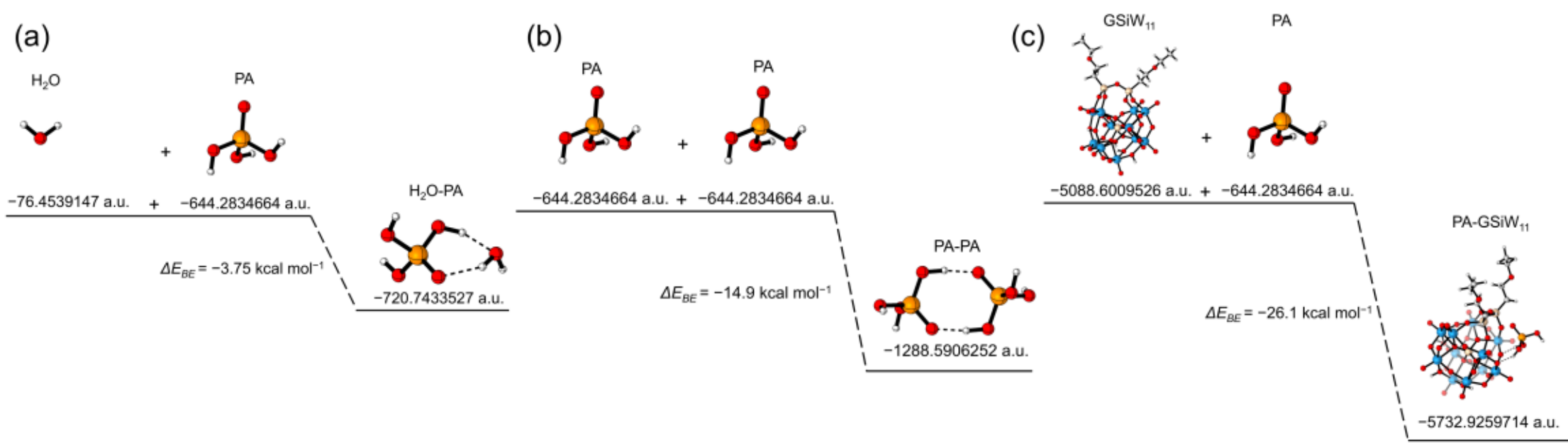

Figure S6. Binding energies (ΔE_{BE}) for (a) PA-H_2O, (b) PA-PA, and (c) PA-$GSiW_{11}$ complexes. The energies of the isolated components and the complexes are given in atomic units (a.u.), and the corresponding binding energies (ΔE_{BE}) are reported in kcal mol^{-1}.

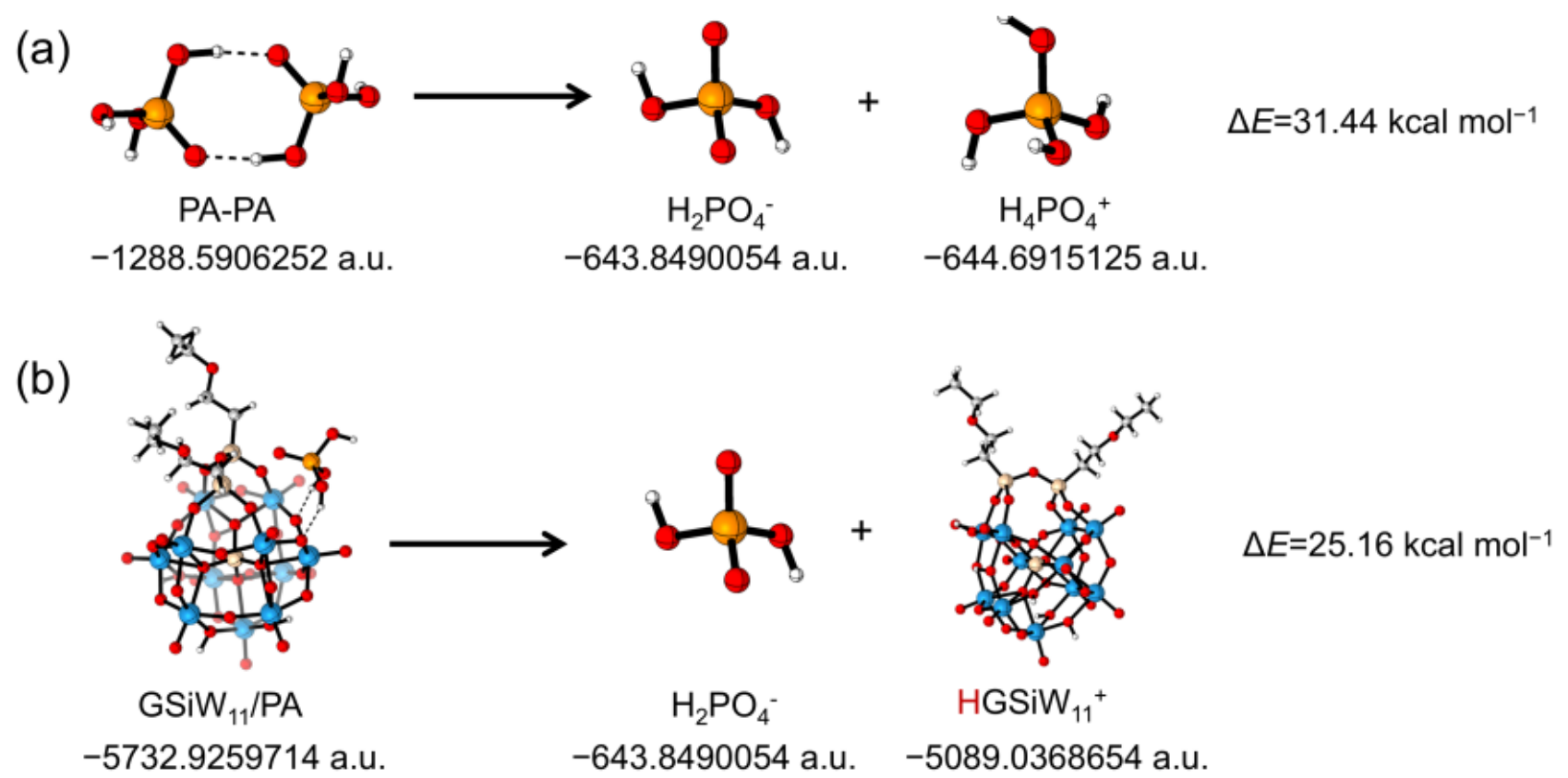

Figure S7. Calculated dissociation energies for (a) the PA-PA complex dissociating into $H_2PO_4^-$ and $H_4PO_4^+$, and (b) the PA-GSiW11 complex dissociating into $H_2PO_4^-$ and $HGSiW_{11}^+$. The energies of the complexes and isolated components are given in atomic units (a.u.), and the dissociation energies are reported in kcal mol^{-1}.

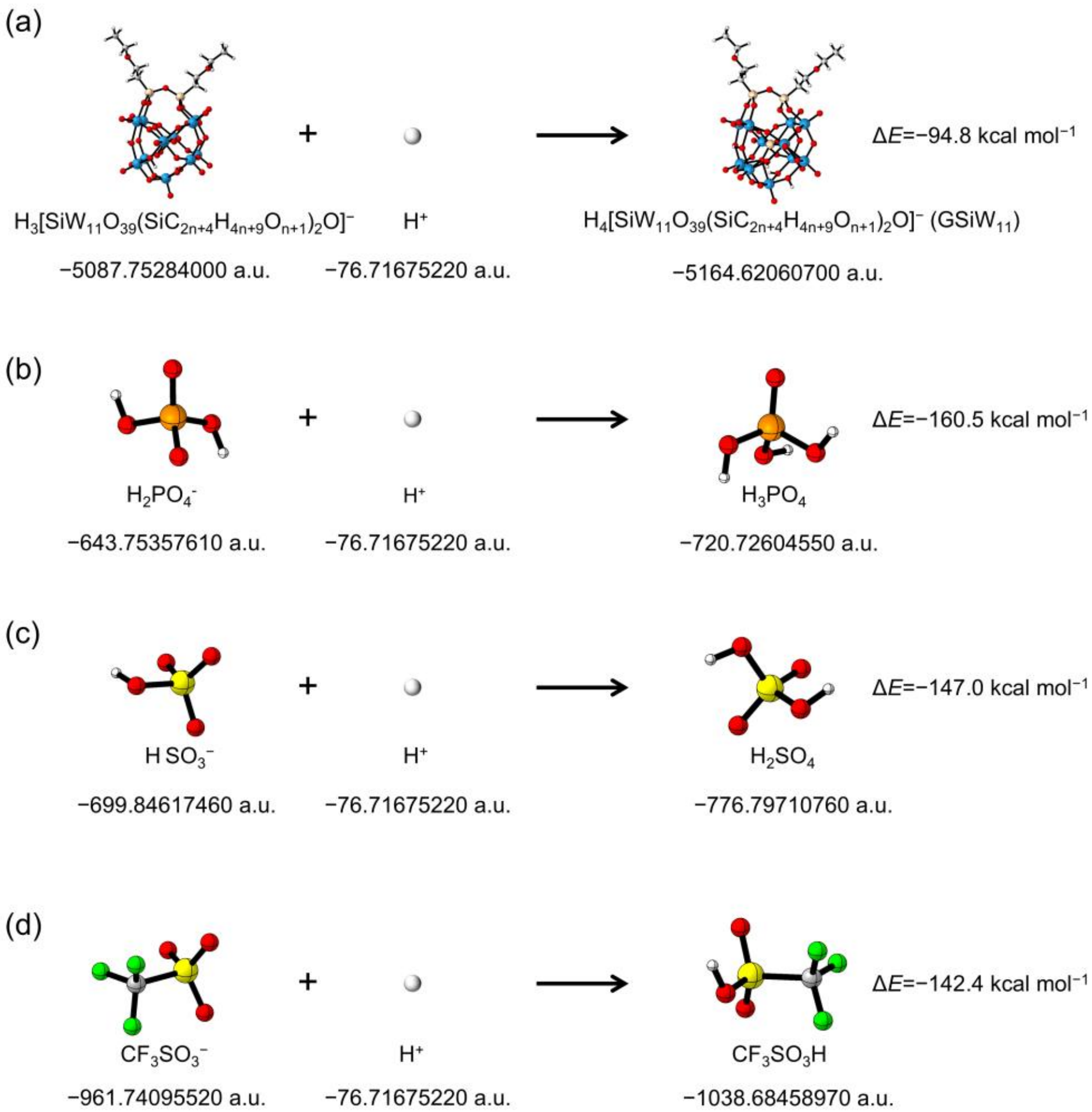

Figure S8. Calculated proton affinities of (a) $H_3[SiW_{11}O_{39}(SiC_{2n+4}H_{4n+9}O_{n+1})_2O]^-$, (b) $H_2PO_4^-$, (c) HSO_4^-, and (d) $CF_3SO_3^-$ in the gas phase. The energies of the corresponding species are given in atomic units (a.u.), and the calculated proton affinities are reported in kcal mol^{-1}.

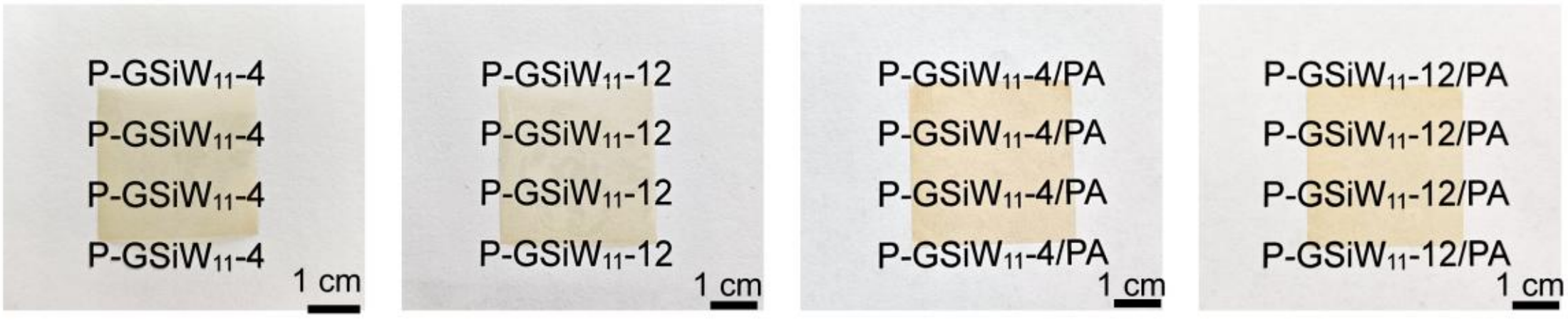

Figure S9. Photographs of P-GSiW$_{11}$-x and P-GSiW$_{11}$-x/PA (x = 4 and 12).

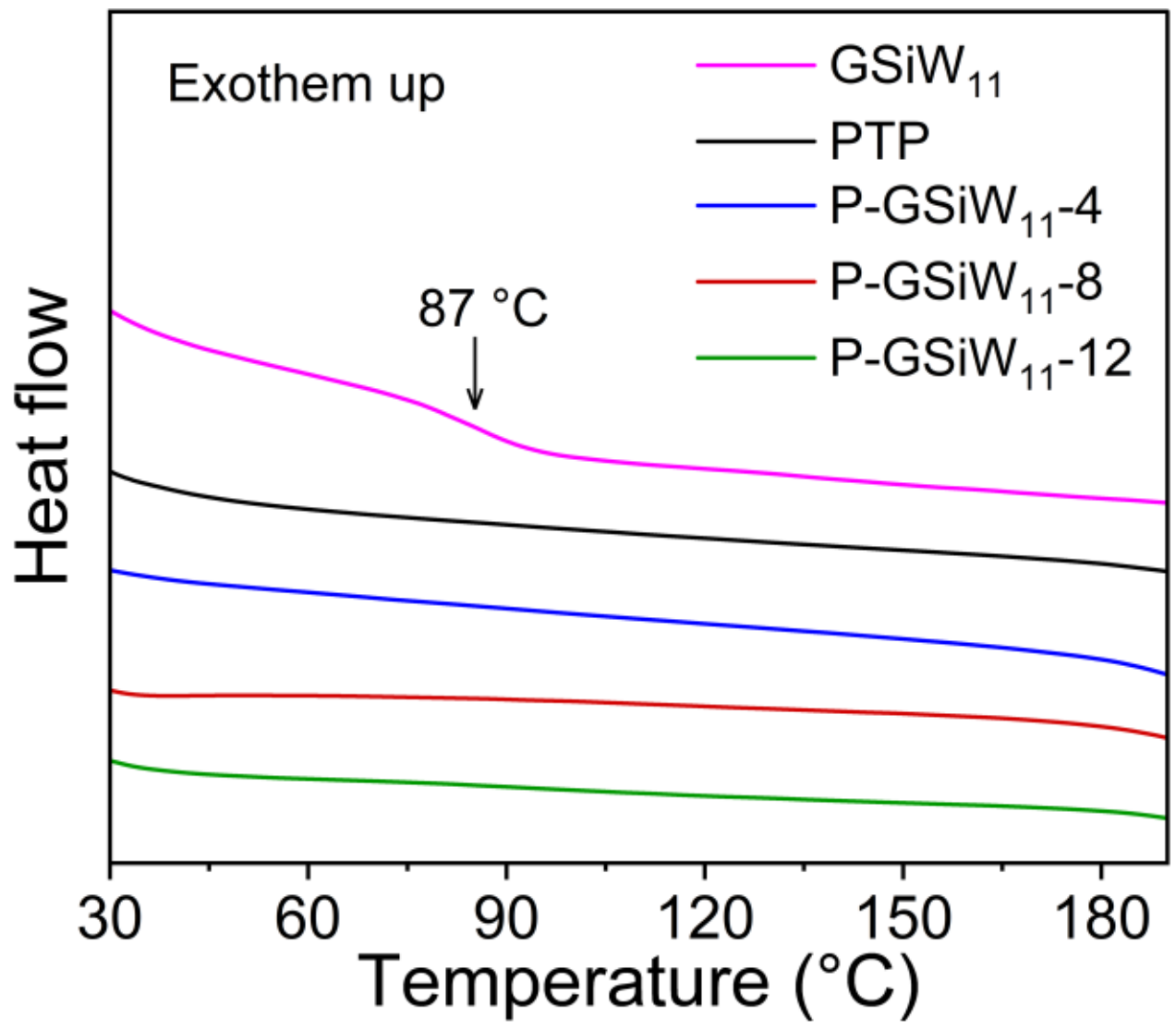

Figure S10. DSC curves of GSiW$_{11}$, PTP and P-GSiW$_{11}$-x (x = 4, 8, and 12).

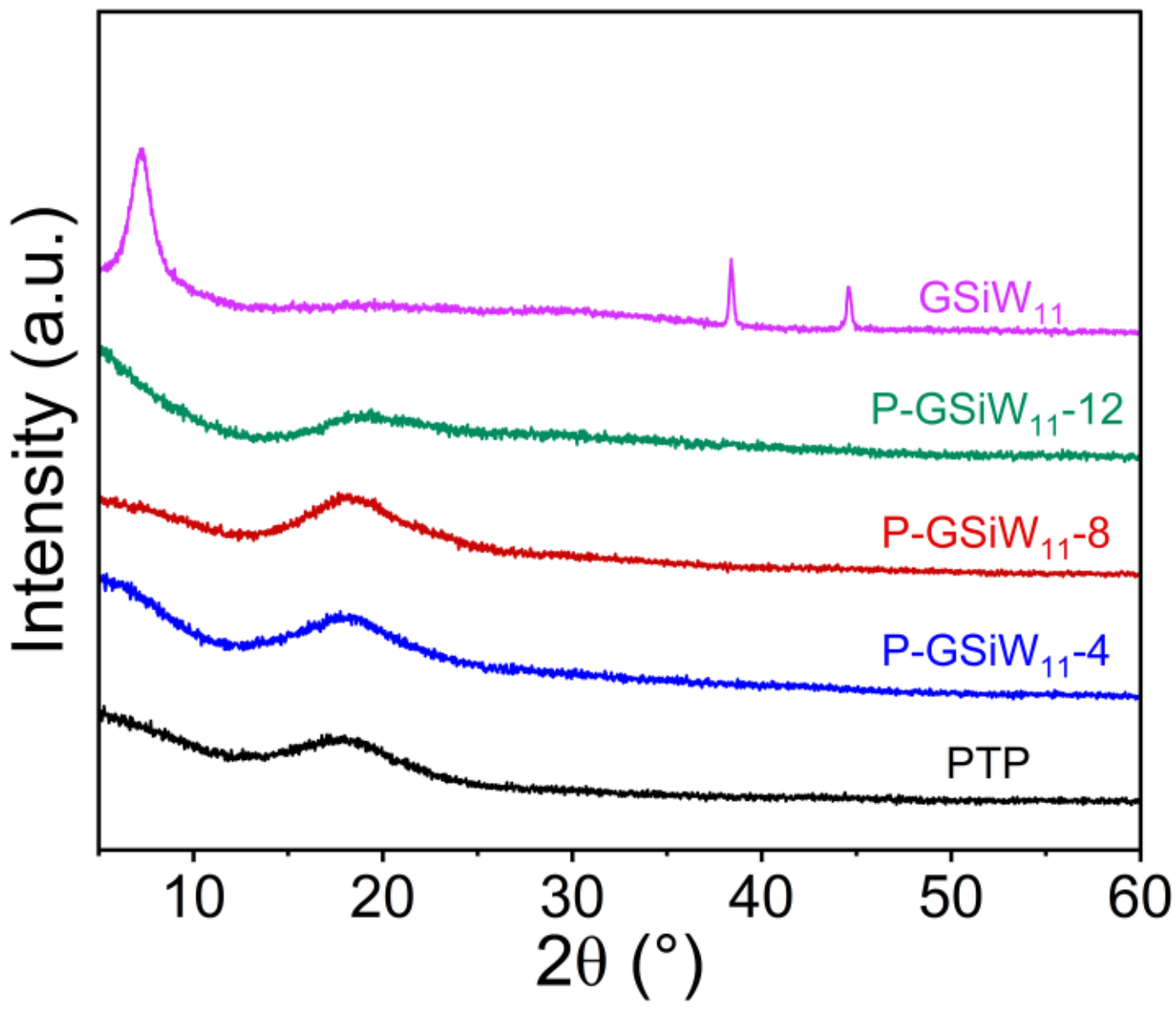

Figure S11. XRD patterns of GSiW$_{11}$, PTP and P-GSiW$_{11}$-x (x = 4, 8, and 12).

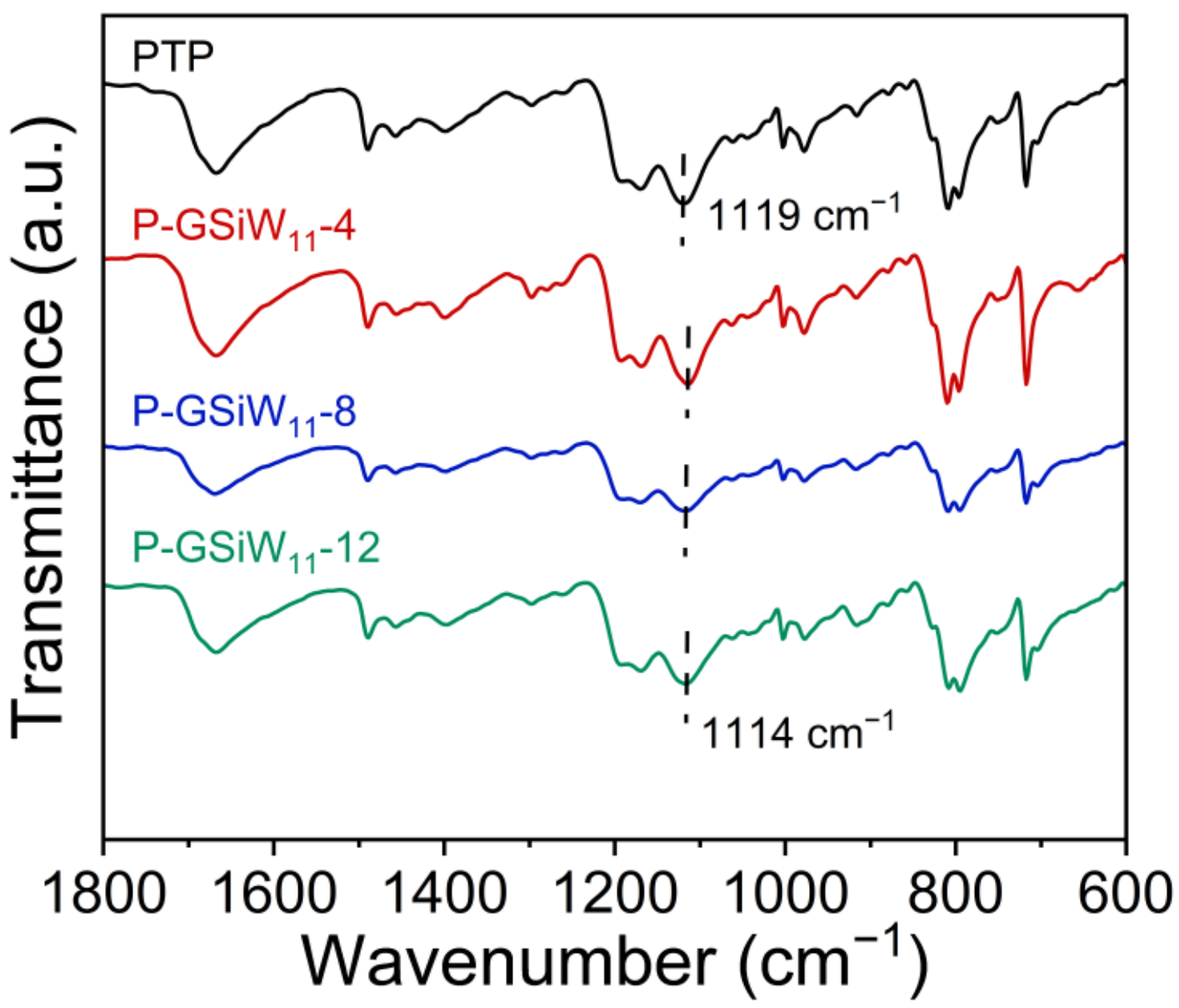

Figure S12. ATR-FTIR spectra of PTP and P-$GSiW_{11}$-*x* (*x* = 4, 8, and 12).

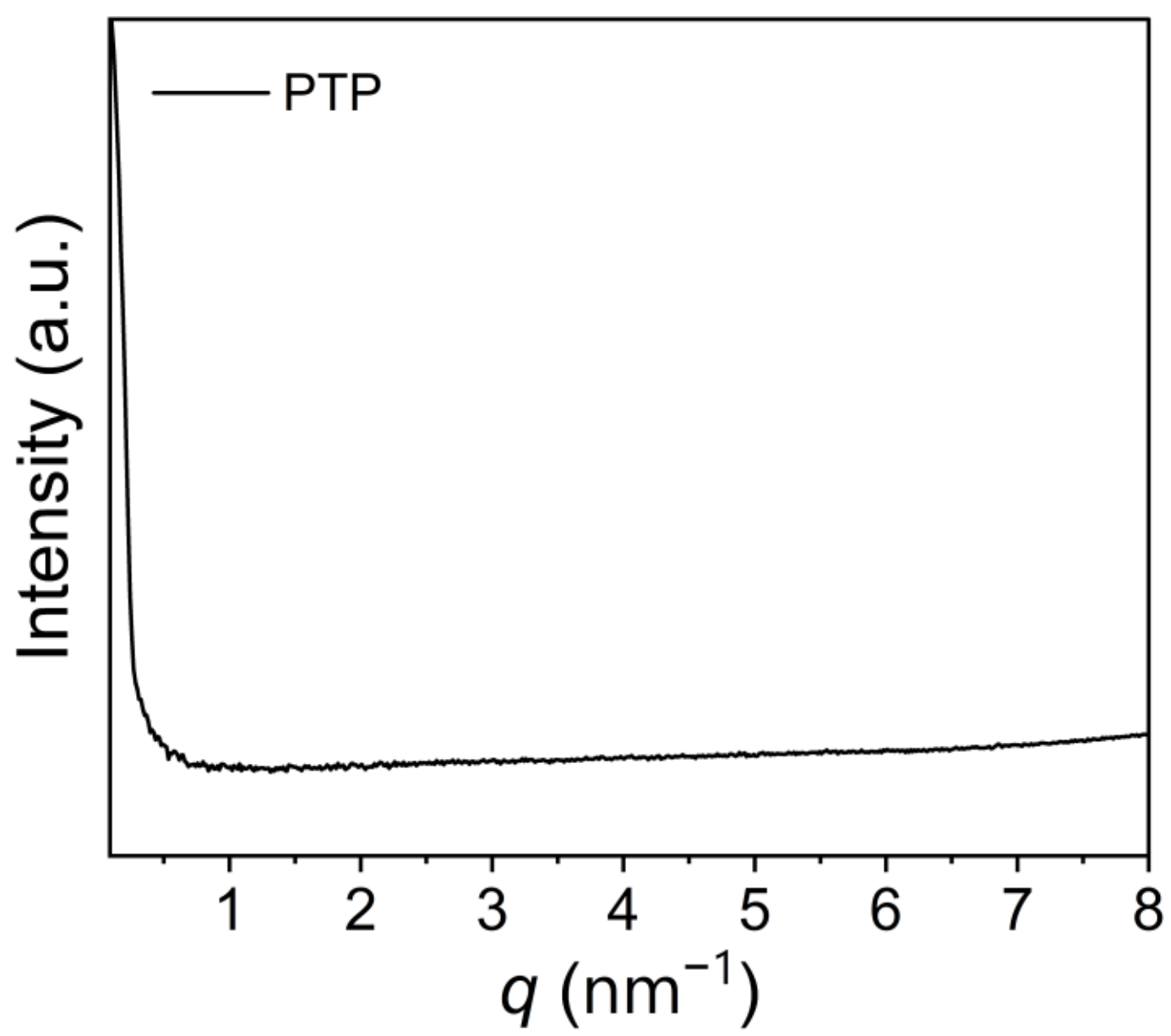

Figure S13. SAXS profiles of PTP.

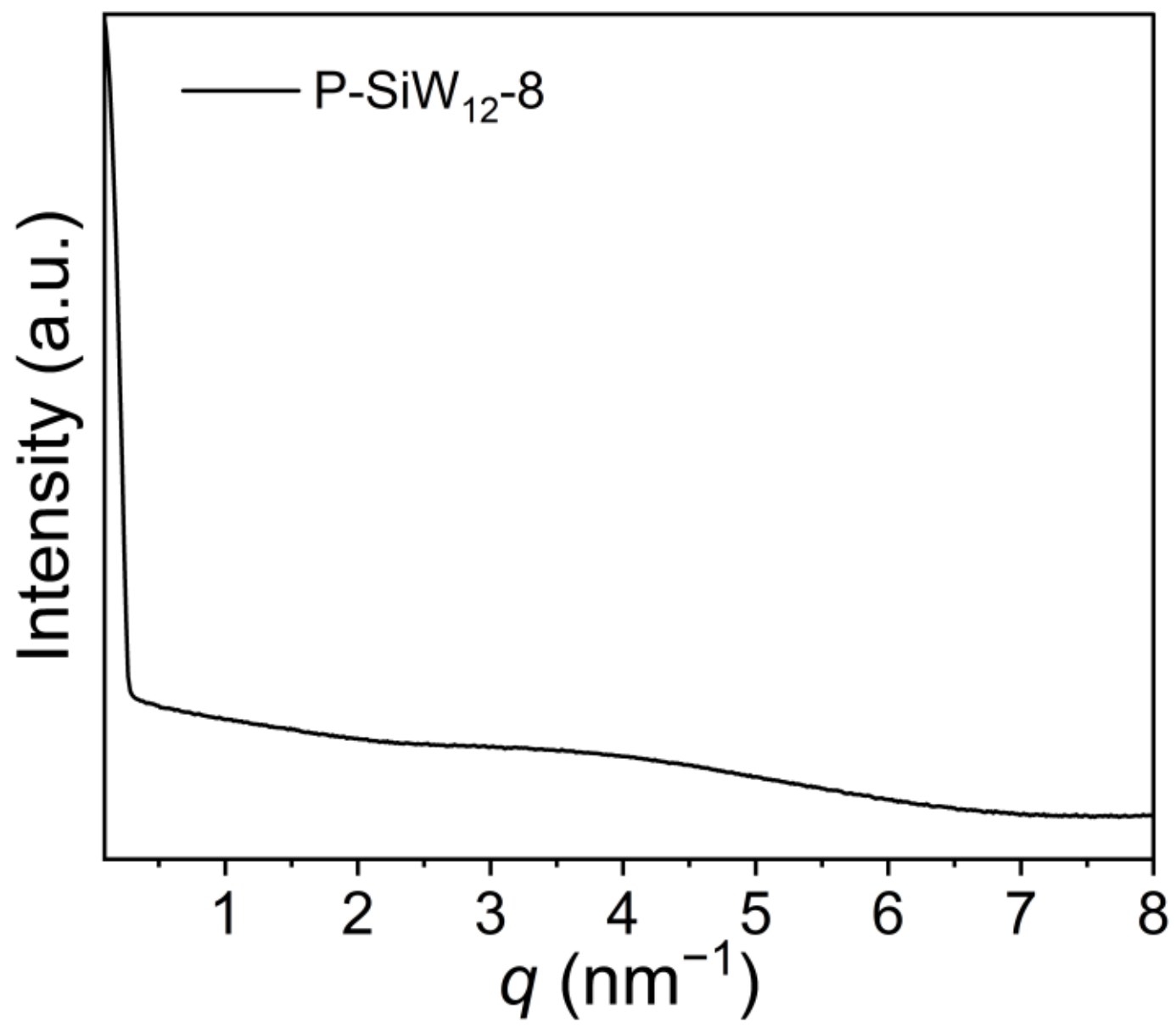

Figure S14. SAXS profiles of P-SiW_{12}-8.

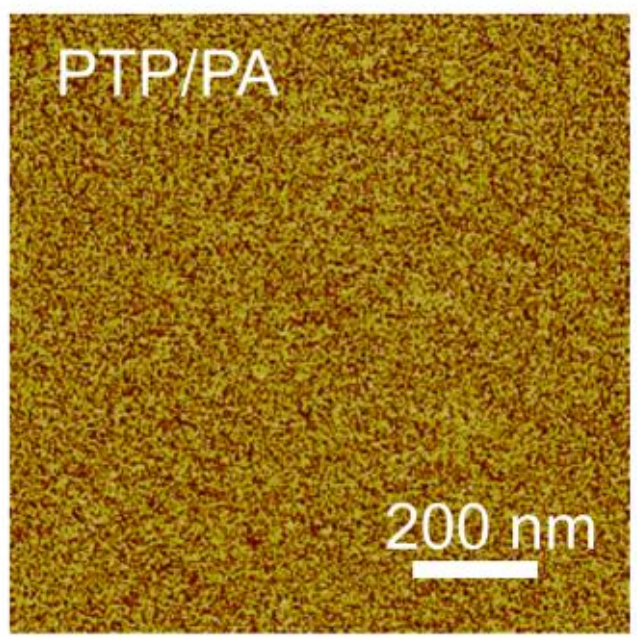

Figure S15. AFM phase images of the thin-sectioned PTP/PA membrane.

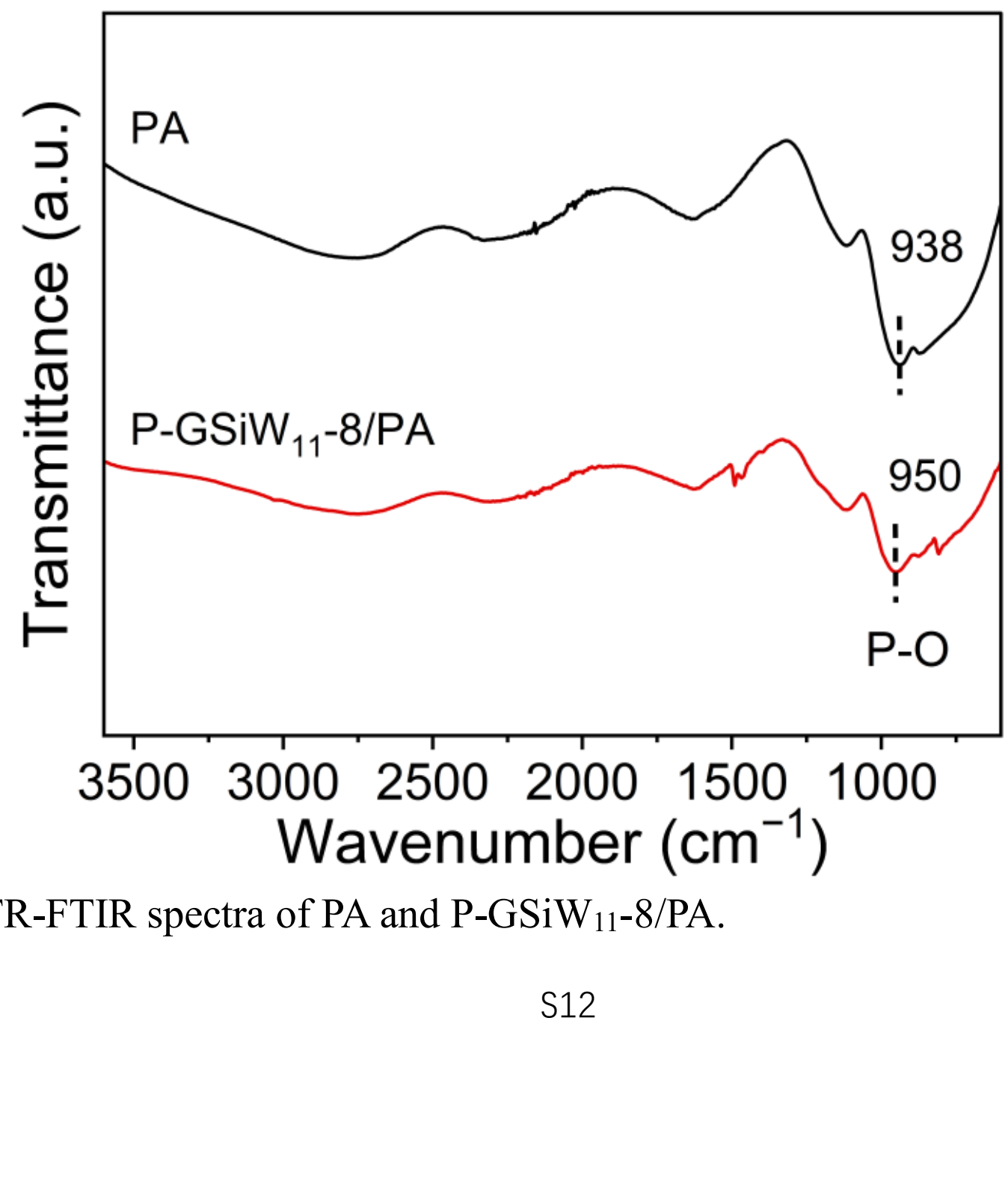

Figure S16. ATR-FTIR spectra of PA and P-$GSiW_{11}$-8/PA.

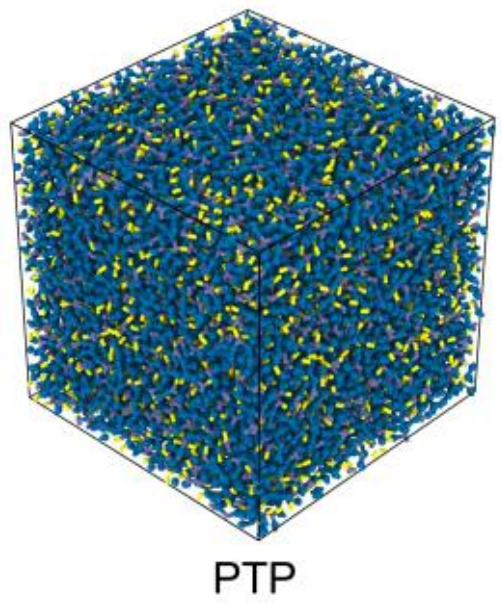

Figure S17. Simulated snapshots of the bulk microphase structures in PTP.

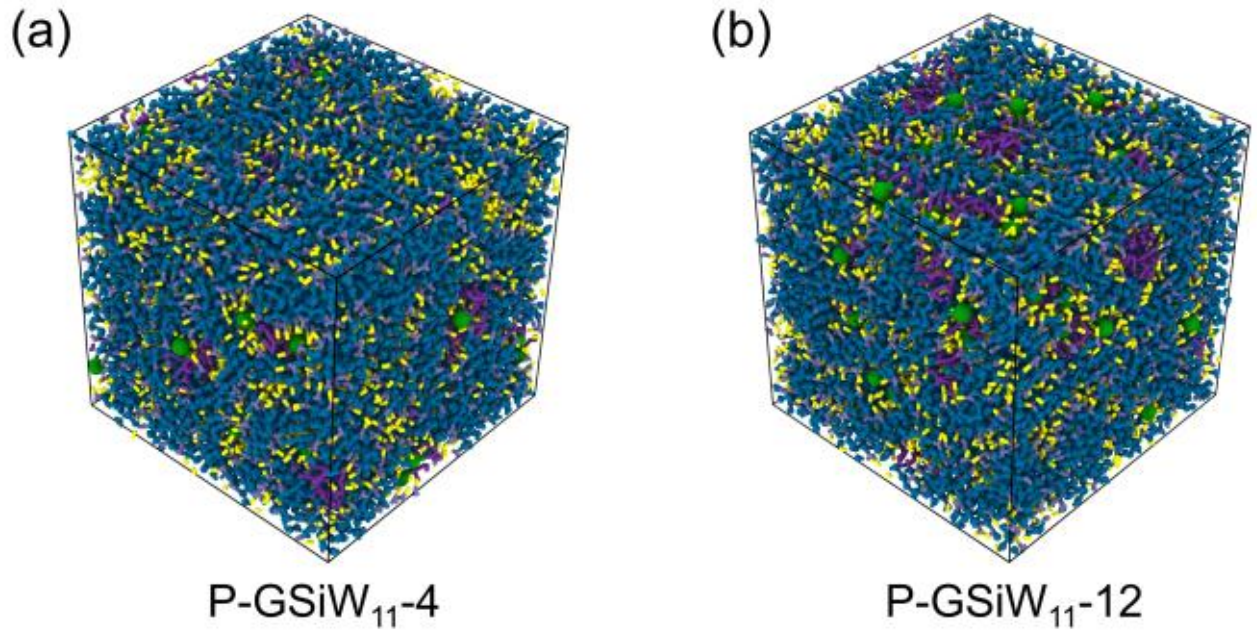

Figure S18. Simulated snapshots of the bulk microphase structures in (a) P-GSiW$_{11}$-4 and (b) P-GSiW$_{11}$-12. Before PA doping, all hybrid membranes exhibit similar microphase structures, in which the hydrophilic domains composed of piperidine groups and GSiW$_{11}$ are continuously distributed within the matrix formed by the aromatic PTP backbone, giving rise to a three-dimensional interconnected network-like morphology.

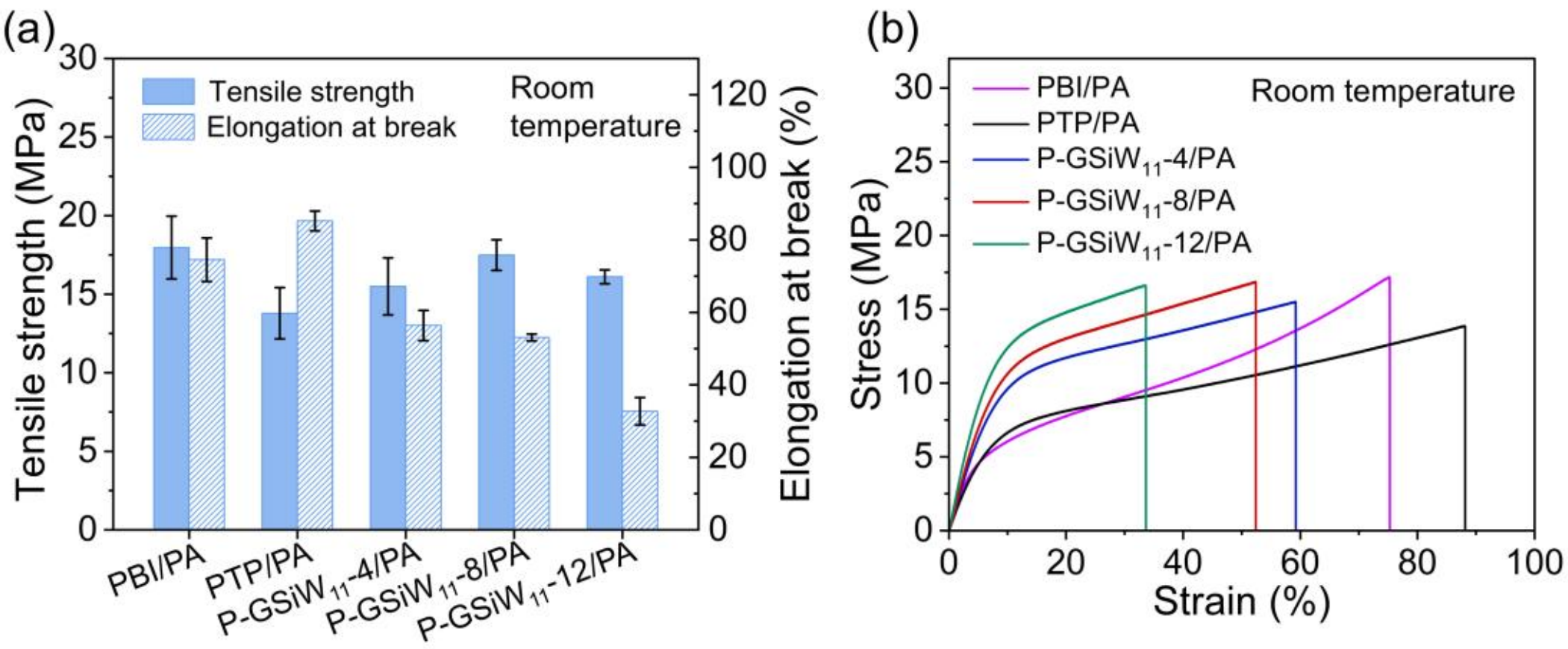

Figure S19. (a) Mechanical properties of PBI/PA, PTP/PA, and P-GSiW$_{11}$-x/PA membranes at room temperature. (b) Stress–strain curves of PBI/PA, PTP/PA, and P-GSiW$_{11}$-x/PA membranes at room temperature (x = 4, 8 and 12).

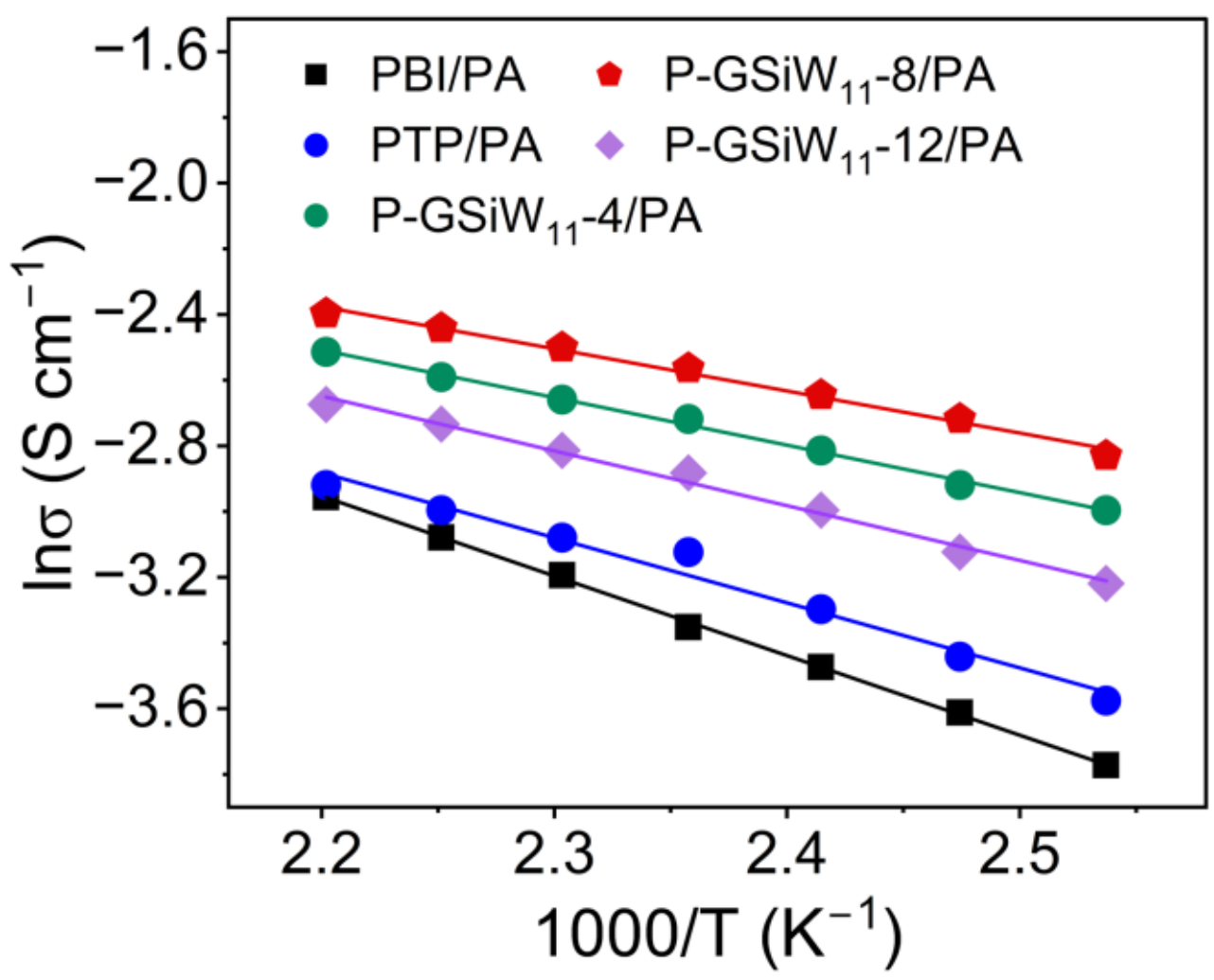

Figure S20. Arrhenius plots of proton conductivities of PBI/PA, PTP/PA and P-GSiW$_{11}$-*x*/PA membranes (x = 4, 8 and 12).

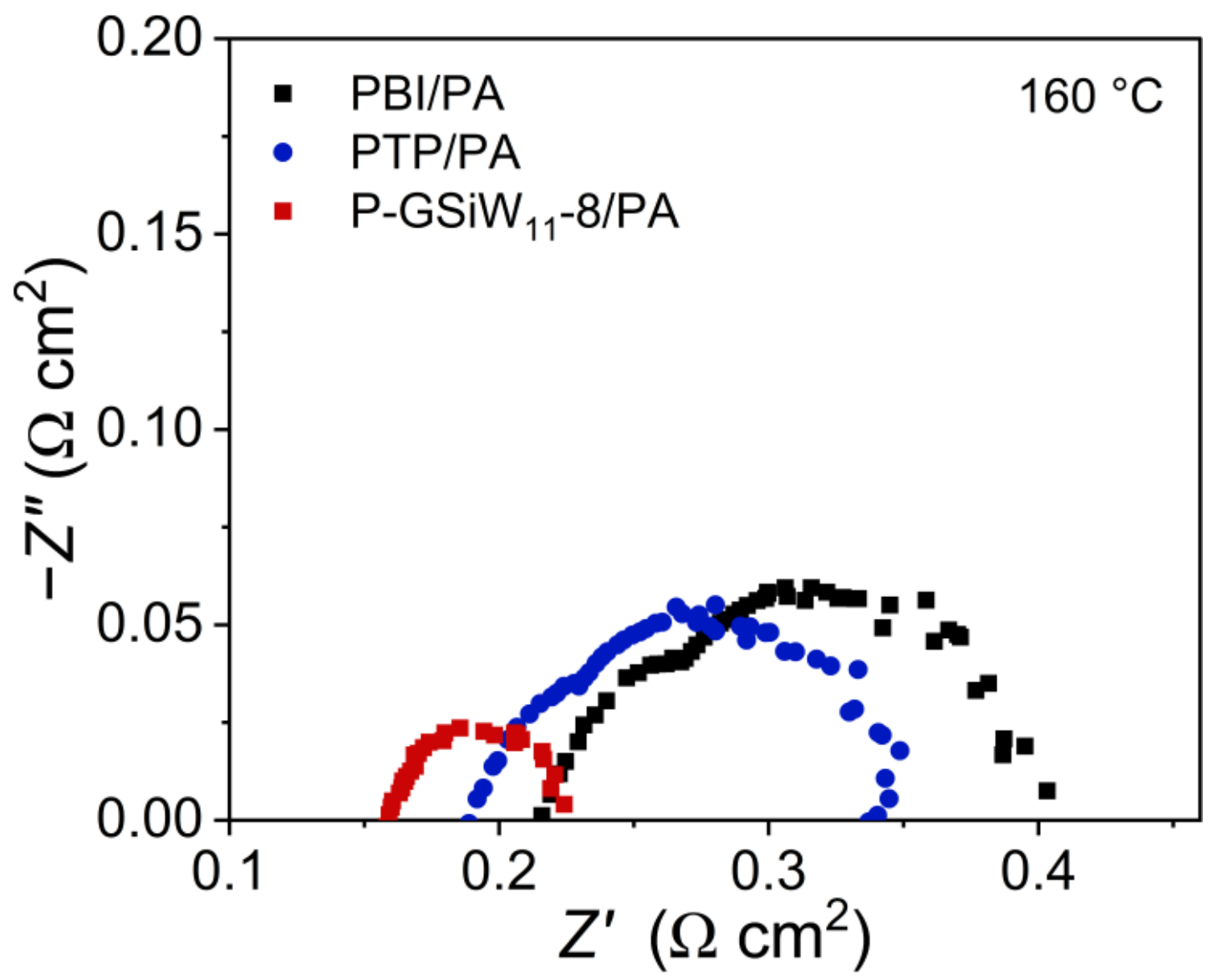

Figure S21. EIS data of PBI/PA, PTP/PA and P-GSiW$_{11}$-8/PA MEAs.

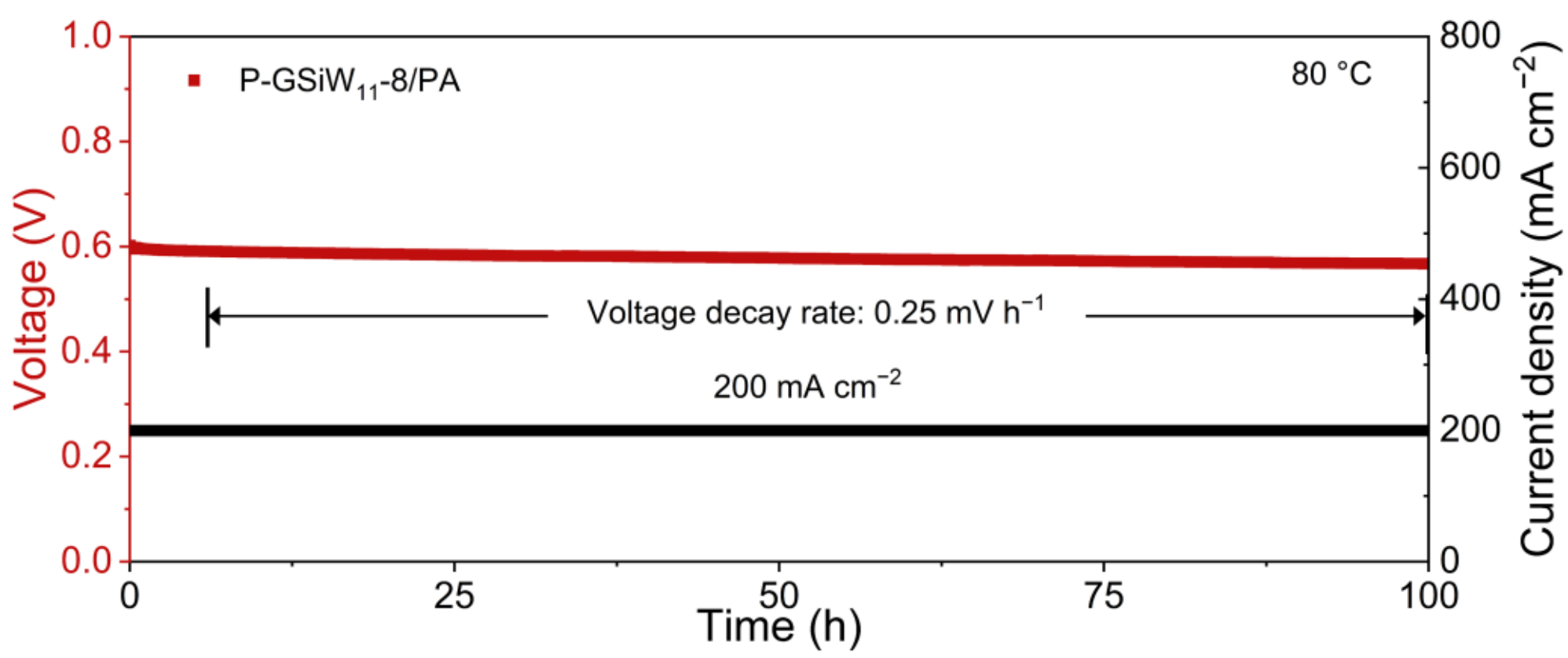

Figure S22. Durability of the single-cell with P-GSiW$_{11}$-8/PA membrane at 80 °C.

7. Supplementary Tables

Table S1. DFT-calculated proton affinities of $H_3[SiW_{11}O_{39}(SiC_{2n+4}H_{4n+9}O_{n+1})_2O]^-$, $H_2PO_4^-$, HSO_4^-, and $CF_3SO_3^-$.

Conjugate base	Proton affinities [kcal mol^{-1}]
$H_3[SiW_{11}O_{39}(SiC_{2n+4}H_{4n+9}O_{n+1})_2O]^-$	−98.4
$H_2PO_4^-$	−160.5
HSO_4^-	−147.0
$CF_3SO_3^-$	−142.4

$H_3[SiW_{11}O_{39}(SiC_{2n+4}H_{4n+9}O_{n+1})_2O]^-$ exhibits the lowest proton affinity among the conjugate bases examined, particularly compared with $H_2PO_4^-$, the conjugate base of H_3PO_4. This low proton affinity indicates relatively weak proton binding in $GSiW_{11}$, facilitating proton sharing with $H_2PO_4^-$ and thereby stabilizing the $H_2PO_4^-$ species.

Table S2. Parameters used in the simulation.

ε	C1	C2	N	Si	G	P1	P2
C1	4.0						
C2	3.0	3.5					
N	2.0	3.5	3.5				
Si	2.0	2.0	5.6	5.0			
G	2.5	2.5	3.0	5.0	4.0		
P1	2.0	2.0	5.6	5.6	5.0	3.5	
P2	2.0	2.0	5.6	5.6	5.0	5.6	3.5

C1 represents the phenyl rings, while C2 and N denote the hydrophobic and hydrophilic segments of the piperidine group, respectively. Specifically, the piperidine ring is divided into two parts, where the segment adjacent to the polymer backbone is defined as the hydrophobic part, and the segment containing the nitrogen atom is regarded as the hydrophilic part. Si represents the SiW clusters in $GSiW_{11}$, G denotes the PEG chains in $GSiW_{11}$, P1 corresponds to phosphoric acid molecules, and P2 represents dihydrogen phosphate ions.

Table S3. PA uptake, proton conductivity and fuel cell performance of P-$GSiW_{11}$-8/PA and reported membranes.

Membranes	PA uptake [wt%]	Conductivity [mS cm^{-1}]	Peak power density [mW cm^{-2}]	Pt load [mg cm^{-2}]	Temperature [°C]	Ref.
P-$GSiW_{11}$-8/PA	125	82	1130	1.0	160	This work
5PA@HNTs/g-ABPBI	256.8	63	354	0.6	160	S20
QPAF-225-10	278	174	847	0.3	200	S21

4 %-CL-OPBI	335.8	121.5	1109.1	0.6	160	S22
PDTPI-6:4	367.7	240	643	0.5	200	S23
p-OPBI-ATMP	156	112	980	1.2	160	S24
PSBI-IM/PA	216.82	40	615	0.6	160	S25
CBOPBI@MOF	126	78	736	1.0	160	S26
PBI-VED-40%	380	86.4	977	0.6	160	S27

8. References

S1. A. Téazéa, G. Hervéa, R. G. Finke and D. K. Lyon, *Inorg. Synth.*, 1990, **27**, 85-96.

S2. H. Bai, H. Peng, Y. Xiang, J. Zhang, H. Wang, S. Lu and L. Zhuang, *J. Power Sources*, 2019, **443**, 227219.

S3. H. He, Y. Zhu, T. Li, S. Song, L. Zhai, X. Li, L. Wu and H. Li, *ACS Nano*, 2022, **16**, 19240-19252.

S4. T. Li, P. Zuo, S. Song, S. Chai and H. Li, *J. Membr. Sci.*, 2026, **749**, 125434.

S5. K. Malek, M. Eikerling, Q. Wang, T. Navessin and Z. Liu, *J. Phys. Chem. C*, 2007, **111**, 13627-13634.

S6. S. J. Marrink, H. J. Risselada, S. Yefimov, D. P. Tieleman and A. H. de Vries, *J. Phys. Chem. B*, 2007, **111**, 7812-7824.

S7. B. Liu, B. Hu, J. Du, D. Cheng, H.-Y. Zang, X. Ge, H. Tan, Y. Wang, X. Duan, Z. Jin, W. Zhang, Y. Li and Z. Su, *Angew. Chem. Int. Ed.*, 2021, **60**, 6076-6085.

S8. C. Sagui and T. A. Darden, *Ann. Rev. Biophys.*, 1999, **28**, 155-179.

S9. Y.-L. Zhu, H. Liu, Z.-W. Li, H.-J. Qian, G. Milano and Z.-Y. Lu, *J. Comput. Chem.*, 2013, **34**, 2197-2211.

S10. Y.-L. Zhu, D. Pan, Z.-W. Li, H. Liu, H.-J. Qian, Y. Zhao, Z.-Y. Lu and Z.-Y. Sun, *Mol. Phys.*, 2018, **116**, 1065-1077.

S11. M. J. Frisch, G. W. Trucks, H. B. Schlegel, G. E. Scuseria, M. A. Robb, J. R. Cheeseman, G. Scalmani, V. Barone, G. A. Petersson, H. Nakatsuji, X. Li, M. Caricato, A. V. Marenich, J. Bloino, B. G. Janesko, R. Gomperts, B. Mennucci, H. P. Hratchian, J. V. Ortiz, A. F. Izmaylov, J. L. Sonnenberg, Williams, F. Ding, F. Lipparini, F. Egidi, J. Goings, B. Peng, A. Petrone, T. Henderson, D. Ranasinghe, V. G. Zakrzewski, J. Gao, N. Rega, G. Zheng, W. Liang, M. Hada, M. Ehara, K. Toyota, R. Fukuda, J. Hasegawa, M. Ishida, T. Nakajima, Y. Honda, O. Kitao, H. Nakai, T. Vreven, K. Throssell, J. A. Montgomery Jr., J. E. Peralta, F. Ogliaro, M. J. Bearpark, J. J. Heyd, E. N. Brothers, K. N. Kudin, V. N. Staroverov, T. A. Keith, R. Kobayashi, J. Normand, K. Raghavachari, A. P. Rendell, J. C. Burant, S. S. Iyengar, J. Tomasi, M. Cossi, J. M. Millam, M. Klene, C. Adamo, R. Cammi, J. W. Ochterski, R. L. Martin, K. Morokuma, O. Farkas, J. B. Foresman and D. J. Fox, 2016, Gaussian 16 Rev. B.01, Wallingford, CT.

S12. C. Lee, W. Yang and R. G. Parr, *Phys. Rev. B*, 1988, **37**, 785-789.

S13. A. D. Becke, *J. Chem. Phys.*, 1993, **98**, 1372-1377.

S14. P. J. Stephens, F. J. Devlin, C. F. Chabalowski and M. J. Frisch, *J. Phys. Chem.*, 1994, **98**, 11623-11627.

S15. K. S. Thanthiriwatte, E. G. Hohenstein, L. A. Burns and C. D. Sherrill, *J. Chem. Theory Comput.*, 2011, **7**, 88-96.

S16. S. Grimme, J. Antony, S. Ehrlich and H. Krieg, *The Journal of Chemical Physics*, 2010, **132**.

S17. P. J. Hay and W. R. Wadt, *J. Chem. Phys.*, 1985, **82**, 270-283.

S18. D. Andrae, U. Häußermann, M. Dolg, H. Stoll and H. Preuß, *Theor. Chim. Acta*, 1990, **77**, 123-141.
S19. A. V. Marenich, C. J. Cramer and D. G. Truhlar, *J. Phys. Chem. B*, 2009, **113**, 6378-6396.
S20. B. Wang, Z. Ling, Q. Liu, X. Fu, R. Zhang, S. Hu, F. Zhao, X. Li, X. Bao, N. Li and J. Yang, *Adv. Funct. Mater.*, 2026, **36**, e18482.
S21. X. Zong, H. Mi, F. Chen, X. Guan, Y. Liu, W. Hu, N. Li, C. Jiang, Y. Lu, G. Zhu, W. Yan and J. Zhang, *Angew. Chem. Int. Ed.*, 2025, **64**, e202509085.
S22. J. Peng, Y. Xu, X.-z. Fu and L. Wang, *J. Membr. Sci.*, 2026, **738**, 124783.
S23. Q. Wang, L. Sang, G. Gao, J. Guan, H. Yu, J. Zheng, Q. Zhang, G. Qin, S. Li and S. Zhang, *Macromolecules*, 2025, **58**, 8295-8307.
S24. W. Li, W. Liu, J. Zhang, H. Wang, S. Lu and Y. Xiang, *Adv. Funct. Mater.*, 2023, **33**, 2210036.
S25. B. Liu, Q. Liu, Y. Pang, Y. Duan and C. Zhao, *Adv. Funct. Mater.*, 2025, **35**, 2408291.
S26. P. Wang, J. Lin, Y. Wu and L. Wang, *J. Power Sources*, 2023, **560**, 232665.
S27. J. Lin, P. Wang, J. Bin and L. Wang, *Small*, 2024, **20**, 2311767.
